# The Agent Preference in Visual Event Apprehension

**DOI:** 10.1162/opmi_a_00083

**Published:** 2023-06-09

**Authors:** Arrate Isasi-Isasmendi, Caroline Andrews, Monique Flecken, Itziar Laka, Moritz M. Daum, Martin Meyer, Balthasar Bickel, Sebastian Sauppe

**Affiliations:** Department of Comparative Language Science, University of Zurich, Zurich, Switzerland; Center for the Interdisciplinary Study of Language Evolution (ISLE), University of Zurich, Zurich, Switzerland; Department of Linguistics, Amsterdam Centre for Language and Communication, University of Amsterdam, Amsterdam, The Netherlands; Department of Linguistics and Basque Studies, University of the Basque Country (UPV/EHU), Leioa, Spain; Department of Psychology, University of Zurich, Zurich, Switzerland; Jacobs Center for Productive Youth Development, University of Zurich, Zurich, Switzerland; Cognitive Psychology Unit, University of Klagenfurt, Klagenfurt, Austria

**Keywords:** event apprehension, eye tracking, event roles, agents, patients, case marking, Basque, Spanish, brief exposure paradigm

## Abstract

A central aspect of human experience and communication is understanding events in terms of agent (“doer”) and patient (“undergoer” of action) roles. These event roles are rooted in general cognition and prominently encoded in language, with agents appearing as more salient and preferred over patients. An unresolved question is whether this preference for agents already operates during apprehension, that is, the earliest stage of event processing, and if so, whether the effect persists across different animacy configurations and task demands. Here we contrast event apprehension in two tasks and two languages that encode agents differently; Basque, a language that explicitly case-marks agents (‘ergative’), and Spanish, which does not mark agents. In two brief exposure experiments, native Basque and Spanish speakers saw pictures for only 300 ms, and subsequently described them or answered probe questions about them. We compared eye fixations and behavioral correlates of event role extraction with Bayesian regression. Agents received more attention and were recognized better across languages and tasks. At the same time, language and task demands affected the attention to agents. Our findings show that a general preference for agents exists in event apprehension, but it can be modulated by task and language demands.

## INTRODUCTION

To understand the complex reality of everyday life, we need to attend to the events unfolding around us. Events are dynamic interactions that develop over space and time (Altmann & Ekves, 2019; Richmond & Zacks, 2017). A crucial component of events is their participants or event roles, as well as the interaction that binds them. The most basic event roles are the doer of the action (“agent”) and the undergoer to whom the action is done (“patient”). The action in the event is defined by the specific relationship between these two roles (the event type, e.g., *seeing* or *kicking*).

Humans have been proposed to categorize critical information in events using event models (Zacks, 2020). These models are mental representations in memory used to segment the perceived ongoing activity into structured events (Radvansky & Zacks, 2011; Zacks et al., 2007), probably using specialized neural mechanisms (Baldassano et al., 2018; Stawarczyk et al., 2021). Event models are believed to store information on the abstract structure of events, including the event roles.

When exposed to events, humans can extract event role information in a quick and effortless way (Hafri et al., 2018). Hafri et al. (2013) presented participants with events for as short as 37 and 73 ms, and then asked a probe question about the event type, the agent, or the patient. They found that the event type and event roles could be recognized even with the shortest presentation time. Rissman and Majid (2019) review a range of experimental studies with adults, children and infants, and conclude that there is a universal bias to distinguish agent and patient roles.

The agent and patient event roles are asymmetric in their cognitive status, and so far the evidence suggests that agents are more salient. The agent role is characterized by distinguishing perceptual features, such as outstretched limbs (Hafri et al., 2013; Rissman & Majid, 2019). In contrast, the patient role is defined by the lack of these features, resulting in a more diffuse category (Dowty, 1991). When looking at pictures of events, humans tend to inspect agents more thoroughly than patients or other event elements (Cohn & Paczynski, 2013). Furthermore, the agent role is preferentially attended to in all stages of development in humans (Cohn & Paczynski, 2013; Galazka & Nyström, 2016; New et al., 2007), a preference shared with other animals (V. A. D. Wilson et al., 2022). Taken together, the reported evidence suggests that agents take a privileged position in the basic mechanisms of event processing (Dobel et al., 2007; Gerwien & Flecken, 2016; F. Wilson et al., 2011).

This is consistent with how humans attend to scenes in general: In the inspection of real-world scenes, conceptually relevant information guides attention (Henderson et al., 2009, 2018; Rehrig et al., 2020). This happens in a top-down fashion, that is, by higher-order cognitive representations affecting the information uptake. In the inspection of events specifically, the agent is arguably the conceptually most relevant or salient element, and would therefore guide visual attention in a top-down fashion. We use the term “agent preference” to refer to the presumably privileged status of agents in event cognition.

The agent preference finds parallels in other domains, too. When communicating about events, agents occupy privileged positions in how they are expressed. Semantic role categories in language are organized hierarchically, and theories converge on ranking agents the highest in this hierarchy for predicting the morphosyntactic properties of event role expressions (e.g., Bresnan, 1982; Fillmore, 1968; Gruber, 1965; Van Valin, 2006). Agents also play an important role in generating predictions in incremental sentence processing since they tend to be the expected default interpretation for noun phrases (Bickel et al., 2015; Demiral et al., 2008; Haupt et al., 2008; Kamide et al., 2003; Matzke et al., 2002; Sauppe, 2016). When gesturing about events, naïve participants across cultures tend to place the agent first, independently of the word order of their language (Gibson et al., 2013; Goldin-Meadow et al., 2008; Hall et al., 2013; Schouwstra & de Swart, 2014). This mirrors the tendency for placing agents first across languages (Dryer, 2013; Napoli & Sutton-Spence, 2014). In sum, this evidence supports the idea that there is a general preference for agents in cognition.

However, many studies investigating attention to events were not designed to specifically address this cognitive bias. Their findings can therefore only be interpreted indirectly and also might be at least partially confounded by other visual properties. Most studies on event cognition have used non-human and smaller-sized patients (Dobel et al., 2007; Gerwien & Flecken, 2016; Ünal, Richards, Trueswell, & Papafragou, 2021), such as, for example, an event in which a woman cuts a potato. Size, animate motion, and human features attract visual attention (Frank et al., 2009; Pratt et al., 2010; Wolfe & Horowitz, 2017) and this might have biased the attention toward agents in these studies.

So far, two studies have attempted to account for animacy when testing attention allocation in events (Cohn & Paczynski, 2013; Hafri et al., 2018). In these experiments, both agents and patients were human and of similar size. Cohn and Paczynski (2013) measured looking times to the event roles in cartoon strips that were presented frame by frame. They found longer looking times for agents than for patients and argued that the advantage for agents stemmed from them being the initiators of the action. Hafri et al. (2018) also used human agents and patients in their stimuli, but unlike Cohn and Paczynski (2013), they did not find a preference for agents. When viewing event photographs for short durations, participants responded faster to low-level features in patients than in agents. This is potential counter-evidence against the agent preference, opening the possibility that this preference is not a cognitive bias per se, but rather a side effect of an animacy bias, in line with findings from emergent sign languages (Meir et al., 2017). An agent preference could also emerge from conscious decision-making and only in a later time frame when attending to human-human interactions. This would explain why Cohn and Paczynski (2013) found an agent preference in a self-paced task, while Hafri et al. (2018) did not find such a preference when using brief stimulus presentation times and high time pressure. Hence, it remains unknown whether the agent preference operates independently of animacy, and whether it arises in the earliest stages of attending to events.

In the present work, we investigate whether an agent preference in event cognition is detectable in early visual attention. We include both human and non-human patients in the stimuli, as well as patients of different sizes. Following Dobel et al. (2007) and Hafri et al. (2018), we focus on the apprehension phase of processing events. We define event apprehension as the phase in which the gist of an event is obtained, covering approximately up to the first 400 ms after seeing an event picture (Griffin & Bock, 2000). We chose the apprehension phase specifically because it captures the earliest and most spontaneous allocation of visual attention. During apprehension, agent and patient roles are extracted spontaneously and independently of an explicit goal, that is, also when the task requires only the extraction of low-level features (such as color) and does not encourage the processing of event roles (Hafri et al., 2013, 2018). If there is a general agent preference in event cognition, it should be detectable already in this phase.

To target event apprehension, we adapted the brief exposure paradigm from Dobel et al. (2007) and Greene and Oliva (2009). In this paradigm, pictures of events are presented for only very short periods of time, typically between 30 and 300 ms, depending on the screen position in which the picture appears (Dobel et al., 2007, 2011). Because planning and launching a saccade already takes between 150 and 200 ms (R. H. S. Carpenter & Williams, 1995; Duchowski, 2007; Pierce et al., 2019), viewers need to make quick decisions about what to look at. These decisions are arguably based on prior information and task-related knowledge (Gerwien & Flecken, 2016).

As well as probing for an agent preference in the earliest time window of attention, we also test whether this preference persists across different languages and task configurations. Indeed, the agent preference is likely to interact with other top-down cues that guide visual attention, such as knowledge of the event, prior experiences, and task demands (e.g., Summerfield & de Lange, 2014). An important task is producing sentences in a specific language, because language can exert a top-down influence on the way events are inspected (Norcliffe & Konopka, 2015). For example, Norcliffe et al. (2015) used a picture description experiment with speakers of languages with different word order (subject-initial vs. verb-initial), and showed that speakers of verb-initial sentences in Tzeltal (Mayan) prioritized attending to verb- or action-related information over agents (cf. also Sauppe et al., 2013).

In our study, we test whether an agent preference persists across two languages that mark agents differently, namely Basque and Spanish. Basque has a case marker (conventionally known as ‘ergative’) specifically for agent noun phrases, while Spanish does not have an agent-specific case marker. This means that in Basque, agentive subjects are overtly marked (*-k* in the examples in 1), and non-agentive subjects are left unmarked. In Spanish, by contrast, both agent and patient subjects are treated alike, independently of their event role (carrying the unmarked nominative case, similar to English and German). This difference is illustrated in the following sentences in Basque and Spanish. In Basque, only agentive subjects (1a–b in contrast to 1c) receive the ergative case marker. In Spanish (2), instead, all subjects are unmarked (no ergative case marking).

(1)  Basque^1^
  a.  Emma-**k**   mahaia  altxatu  du
      Emma-erg  table   lift   aux
      ‘Emma lifted the table.’
  b.  Emma-**k**   borrokatu  du
      Emma-erg  fight    aux
      ‘Emma fought.’
  c.  Emma-∅   iritsi  da
      Emma-nom  arrive  aux
      ‘Emma arrived.’
(2)  Spanish
  a.  Emma-∅   ha levantado la mesa
      Emma-nom  aux lift   det table
      ‘Emma lifted the table.’
  b.  Emma-∅   ha  luchado
      Emma-nom  aux fight
      ‘Emma fought.’
  c.  Emma-∅   ha llegado
      Emma-nom  aux arrive
      ‘Emma arrived.’

Given this case marking system, speakers must commit to the agentivity of the subject noun phrase early on when planning a sentence in Basque, because they need this information to decide on its case marker (Egurtzegi et al., 2022; Sauppe et al., 2021). This may increase the need to search for agents in events, especially in languages such as Basque, where agency is the critical feature. Hence, the tendency to inspect agents might increase when planning sentences in Basque due to the demands imposed by case marking. In contrast, for Spanish speakers, agent-related information is not necessary to plan the subject argument of the sentence, because the case marking will not be affected. This means that they could defer making a decision for building a description of event roles to a later point in time and thus maintain more flexibility (Bock & Ferreira, 2014; F. Ferreira & Swets, 2002; V. S. Ferreira, 1996).

In our experiments, we tested native Basque and Spanish speakers in an event description task to investigate whether the agent preference persists across these two different case marking settings. This way, we explored whether an agent preference arises in a language production task, independently of language-specific grammatical features.

In addition, we also tested Basque and Spanish participants in a task that does not require sentence planning. Most previous studies that provide evidence for an agent preference involved a task that required participants to describe events (Gerwien & Flecken, 2016; Sauppe & Flecken, 2021). Given that most languages are agent-initial, it is possible that general sentence planning mechanisms give rise to the agent preference. In our experiments, we introduced a task manipulation to explore whether an agent preference emerges also in the absence of sentence planning demands.

Hence, participants in our experiments undertook two tasks: after being presented an event photograph for 300 ms, they either produced a sentence to describe the event (the *event description* task) or decided whether a probe picture matched an event participant from the briefly presented target picture (the *probe recognition* task). The event description task required the linguistic encoding of the event with the corresponding language-specific differences between the Basque-and Spanish-speaking groups. By contrast, the probe recognition task only demanded selective attention to event roles, with no linguistic response. It is still possible that participants covertly recruited or activated language in the probe recognition task (Ivanova et al., 2021), but this task did not require any sentence planning, which probably decreased the activation or use of language.

In Experiment 1, Basque and Spanish speakers participated on the internet and we measured their accuracy and reaction times for each event role in both tasks. In Experiment 2, participants were tested in a laboratory setting, and we used eye tracking to record the eye gaze to the event pictures during the brief exposure period. First fixations have been argued to closely reflect the processes underlying event apprehension (Gerwien & Flecken, 2016), as viewers collect parafoveal information on the event structure and use it to decide on the location of their first fixation to the picture. In comparison, the accuracy and reaction time of the response (the verbal description or the decision on the probe) reflect the outcome of the apprehension stage together with additional cognitive processes, such as memory, post-hoc reasoning, and judgment processes demanded by the task (Firestone & Scholl, 2016). Nevertheless, accuracy and reaction times contain valuable information on the apprehension phase (Hafri et al., 2013, 2018). Thus, we used fixations to pictures, accuracy, and reaction times as three different measures of participants’ attention allocation and information uptake during event apprehension. This procedure allows us to tackle two research questions: Is there an agent preference in attention patterns in visual apprehension? Does this preference persist across different language and task configurations? Based on the findings presented by Cohn and Paczynski (2013), we predicted that if there is a general agent preference in cognition, it should already be detectable in the earliest and most spontaneous stage of attention allocation. In the current experiments, agents thus should receive more visual attention than other event elements across both languages and tasks tested.

## EXPERIMENT 1

### Methods

#### Participants.

Native speakers of Basque (*N* = 90) and Spanish (*N* = 88) participated in an online study.^2^ Social media were used to advertise the study and recruit participants, and monetary prizes were raffled for participation. All participants reported that their native language was still the most common or one of the most common languages in their daily life at the time of participation. The experiment was approved by the ethics committees of the Faculty of Arts and Social Sciences of the University of Zurich (Approval Nr. 19.8.11) and the University of the Basque Country (Approval Nr. M10/2020/007), and all participants gave their informed written consent. All procedures were performed following the ethical standards of the 1964 Helsinki declaration and its later amendments.

#### Materials and procedure.

**Figure 1.**
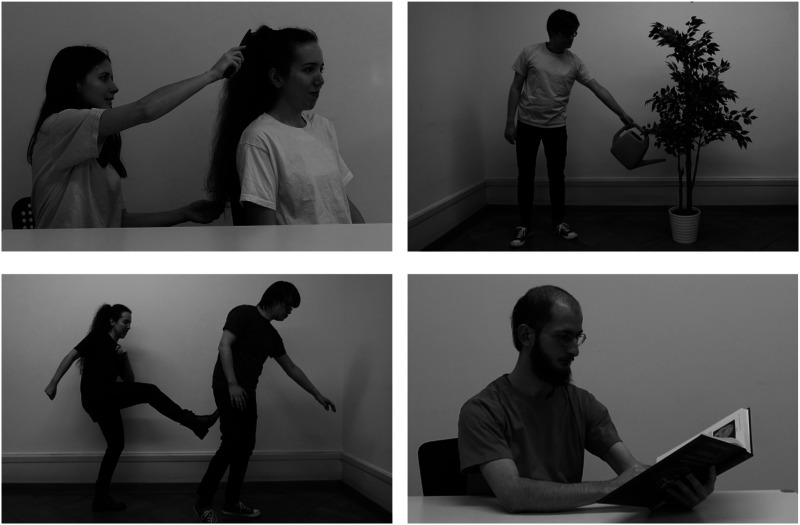
**Four example stimuli, depicting the events “brush”, “water”, “kick” and “read”.** All events were photographed in an indoor setting against a white wall and actors performed the events either standing or sitting at a white table.

Stimuli consisted of 48 photographic gray-scale images depicting transitive, two-participant events with a human agent (see Figure 1 for an example). In half of the events, the patient was human (e.g., with actions such as “hit” or “greet”) and in the other half, an inanimate object was the patient (e.g., with actions such as “wipe” or “hammer”). Ten intransitive events featured a sole participant performing an action and were included as fillers. Within intransitive events, half of them featured an agent-like participant (e.g., “jump”), and the other half a patient-like participant (e.g., “fall down”). A full list of events is presented in Table A1. Photographs depicted the midpoints of events. Static images have been found to spontaneously convey motion information (Guterstam & Graziano, 2020; Kourtzi & Kanwisher, 2000; Krekelberg et al., 2005) so that participants could automatically represent the depicted event sequences as a whole.

The events were portrayed by four different actors (two males, two females). Four versions of each event were photographed, one with each of the four actors as the agent. In the case of events with human patients, the respective actor of the patient was counterbalanced between events, so that the identity of the patient was independent of the identity of the agent. A horizontally mirrored version of each picture was also created to counterbalance the agent’s position across experimental participants. This led to a total of 232 stimulus pictures of 58 events.

The stimulus pictures were distributed over four lists. The set of events was identical across the lists, but with different combinations of actors in agent and patient roles across lists. The lists were used as blocks in the experiment, and participants were randomly assigned two out of the four blocks, with a total of 116 event pictures. The order of events within the blocks was randomized for each participant.

Participants responded to a short demographic questionnaire before the experiment, which included information on gender, age, language acquired from each of their parents, and their most commonly used language. The experiment was programmed in PsychoPy and exported to PsychoJS (Peirce et al., 2019). The experimental sessions were run in full-screen mode on Pavlovia (https://pavlovia.org). Pavlovia offers high temporal resolution (Anwyl-Irvine et al., 2021; Bridges et al., 2020), which ensured that the duration of the stimulus presentation was approximately 300 ms. Mobile phones and tablets were not allowed; participants were directed to the Pavlovia experiment through Psytoolkit (Stoet, 2010, 2017), which enabled blocking access from mobile devices.

**Figure 2.**
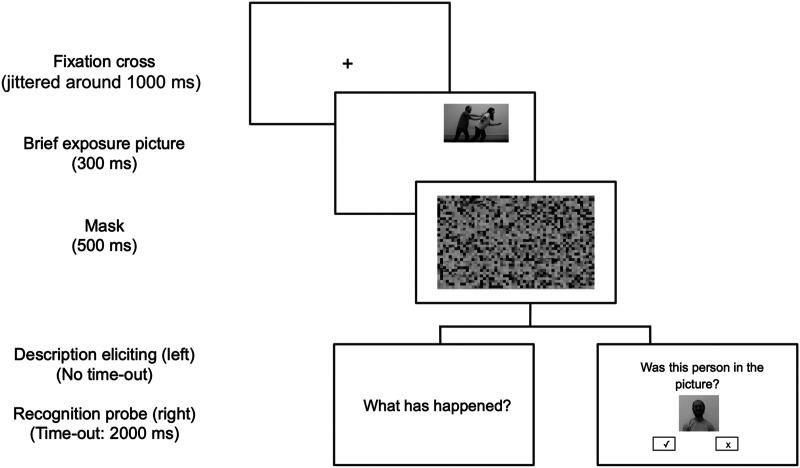
**Trial structure.** The trial procedure was the same until the task and it consisted of a fixation cross, the brief exposure image, and the mask image. Then, in the event description task, the participants were prompted with a question to describe the event image (*Zer gertatu da?* in Basque and *¿Qué ha ocurrido?* in Spanish). In the probe recognition task, a probe image was displayed and participants had to press a button to answer whether that person or object was present in the event.

Trials began with a fixation cross (with a jittered duration time between 800 ms and 1200 ms), followed by the target event picture, which was displayed for 300 ms in one of the four corners of the screen. The orientation (agent left or right) and the picture’s screen position were counterbalanced within the same event types, so that each combination occurred equally often. A mask image appeared immediately after the event picture for a duration of 500 ms (cf. Figure 2). The mask was used to deprive participants of the ability to use their visuospatial sketchpad memory to reconstruct the image (Baddeley, 2007). Following the mask display, participants were prompted to perform an event description or a probe recognition task. The tasks were administered in separate blocks and the order in which the tasks were presented was counterbalanced across participants.

The event description blocks started with the presentation of the four actors and their names. Participants were instructed to learn the four names and use them to describe the events they would see (e.g., “Emma has kicked Tim”). Participants in both languages were instructed to describe events as if they were just completed to elicit sentences in the perfective aspect. This ensured that Basque speakers produced sentences with ergative case marking because there is no ergative marking in the progressive aspect (Laka, 2006). Six example trials were also provided, where sample written descriptions were displayed after the event pictures. The participants also completed six practice trials before proceeding to critical trials.

In the probe recognition task, participants indicated by a button press whether the actor or object in a probe picture had been present in the target event picture or not. The probes showed the agent of the event, the patient of the event, or another actor or object not present in the target picture. Participants were required to respond within 2000 ms, and six practice trials with longer time-outs (7000 ms, down to 3000 ms) were included prior to critical trials.

No feedback was provided for the practice trials in either of the tasks. Figure 2 provides a graphical representation of the trial structure. The experiment sessions lasted approximately 30 minutes. All instructions were provided in Basque or Spanish, respectively.

#### Data processing and analyses.

Nine participants who reported not speaking the same language with both parents were excluded. Data from two participants were lost due to technical errors in data saving. In total, data from 84 native Basque speakers (age range = 18–66 years, mean age = 31.4 years, *SD* = 11.4 years, 55 female) and 82 native Spanish speakers (age range = 18–68 years, mean age = 33.1, *SD* = 11.7 years, 48 female) were available for analysis.

Following the previous literature on the brief exposure paradigm (Dobel et al., 2007; Hafri et al., 2013), we relied on behavioral measures (task specificity, accuracy, and reaction time) as possible windows to event apprehension. In the event description task, written responses largely followed the agent-patient word order patterns, canonical in both languages (see details in Table C1). A native speaker of Basque (A.I.-I.) coded agent, patient, and action specificity for each response, specifying whether the description was specific, general, or inaccurate. We considered answers as *specific* if the name of the event role participant (“Emma”) or object (“bowl”) was correct and as *general* if the description contained correct general features, such as gender or category (e.g., “Lisa” or “One girl” for “Emma”, “pan” for “bowl”). The descriptions that were incorrect (“Tim” instead of “Emma”) or uninformative (“someone”) were coded as *inaccurate*. Event description trials were excluded from analysis if the intended target verb and event roles were inverted (e.g., “Emma has listened to Lisa” instead of “Lisa has shouted to Emma”), if the description was reciprocal (e.g., “Emma and Lisa have shouted to each other”) or if the sentence was not described in perfective aspect (in total, 8% of all event description trials).

For the probe recognition task, we analyzed the trials in which the probes matched either the agent or the patient of the previous event picture (half of all probe recognition trials). The other half of the probe recognition trials showed a foil that was not present in the event picture. We included these foils to ensure that the number of trials requiring a “true” or a “false” answer was balanced, but they were not informative about event role-related accuracy and hence were not included in the analyses. In addition, trials without responses and trials with response times shorter than 200 ms or 2.5 standard deviations longer than the mean were excluded (in total, 6.5% of all probe recognition trials).

**Figure B1.**
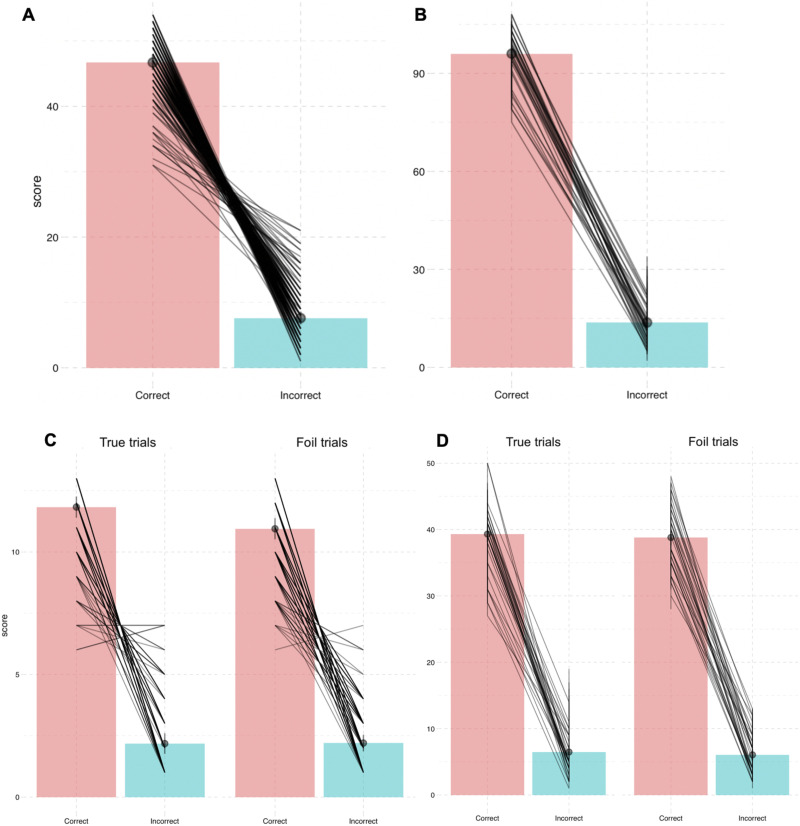
**Accuracy in the probe recognition task for the whole set of trials (including foil trials) in Experiment 1 (A) and Experiment 2 (B).** Figures in the bottom row show accuracy split by true and foil trials for Experiment 1 (C) and Experiment 2 (D). True trials were answered correctly by pressing the “yes” button, and foil trials were correctly answered by pressing the “no” button. In all figures, black dots and colored bars represent the average number of correct and incorrect trials, respectively; lines represent the proportions from individual participants. The direction of individual lines shows that all participants performed above chance.

Participants were additionally excluded from analyses if they performed with overall low accuracy, separately for each task. For the event description analyses, three participants who had less than 60% specific agent answers and five participants who had less than 50% trials remaining after applying the other exclusion criteria were excluded. For the probe recognition analyses, we excluded four participants who had less than 50% trials left after applying the other exclusion criteria or whose overall accuracy was below 60%. We applied these exclusion criteria to ensure that participants in the analyses understood and followed task instructions, and were not performing at chance. For the probe recognition task, we additionally checked that the participants had above-chance accuracy also for the trials with foils (“false” trials). We found that participants correctly rejected foil trials on average in 86% of trials (SE = 0.7%). This ensures that the results from the critical trials (“true” trials) were informative and not driven by a bias to simply answer positively. Figure B1 shows that all participants had above-chance accuracy for the whole set of trials in probe recognition.

On balance, data from 157 participants for the event description task (7071 trials) and from 163 participants (3739 trials) for the probe recognition task were included in statistical analyses.

Statistical analyses were conducted in R (R Core Team, 2022) using hierarchical Bayesian regression through the brms (Bürkner, 2017, 2018) interface to Stan (B. Carpenter et al., 2017). Post-hoc contrasts between the predictor factor levels were extracted with the emmeans package (Lenth, 2020). A cumulative ordinal model with a logit link function was fit to jointly model agent, patient, and action specificity for event description trials (ranking specific > general > inaccurate). For probe recognition analyses, a Bernoulli model with a logit link function was fit to model accuracy in response to agent and patient probes. In both models, event role, language, and their interaction were predictors of interest; identity of the agent role actor, animacy of the patient, and task order were included as nuisance predictors (Sassenhagen & Alday, 2016). Animacy is known to attract visual attention (Frank et al., 2009), and therefore we included it as a covariate to capture its potentially large effects. This ensured that any evidence in favor of the agent preference was not driven by differences between agent and patient animacy in events depicting human-object interactions. We modeled log-transformed reaction times in the probe recognition task with a Gaussian model with an identity link function. Language, event role, and their interaction were the critical predictors, and animacy of the patient, trial accuracy, and task order were included as nuisance predictors. We included random intercepts and slopes for language and event role by participant and by event type in all models. Student-*t* distributed priors (*df* = 5, *μ* = 0, *σ* = 2) were used for the intercept and all population-level predictors in all models. Default priors (Student-*t*, *df* = 3, *μ* = 0, *σ* = 2.5) were used for group-level predictors. In all models, the block number and task order were standardized (*z*-transformed) and all other predictors were sum-coded (−1, 1).

**Figure 3.**
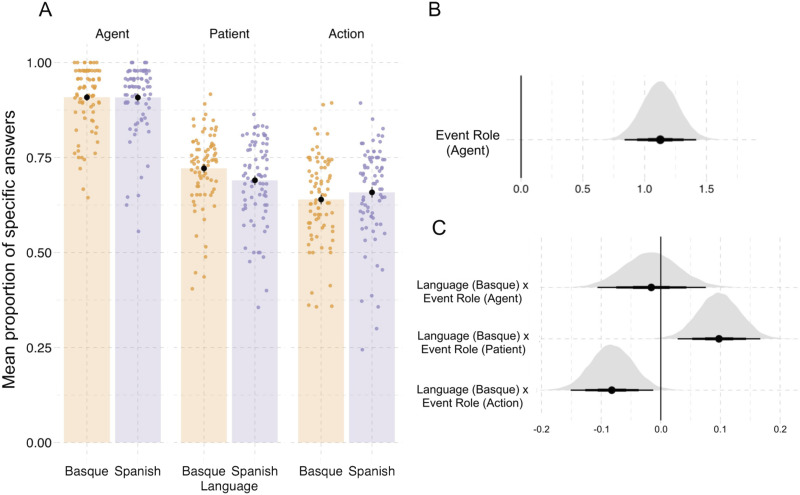
**Results from event description task in Experiment 1.** (A) Specificity in event description, showing the proportion of *specific* answers (in contrast to *general* and *inaccurate* answers). Individual dots represent participant means, black dots represent means of participant means, and error bars on the black dots indicate 1 standard error of the mean. Figure F1 in the Appendix shows fitted values from the Bayesian regression model. (B) Posterior estimates for the predictor event role from the Bayesian regression model (Table D1). (C) Posterior estimates of interactions between each of the event roles (agent, patient, action) and language from the same Bayesian regression model. Black horizontal lines represent the 50%, 80%, and 95% credible intervals.

**Figure 4.**
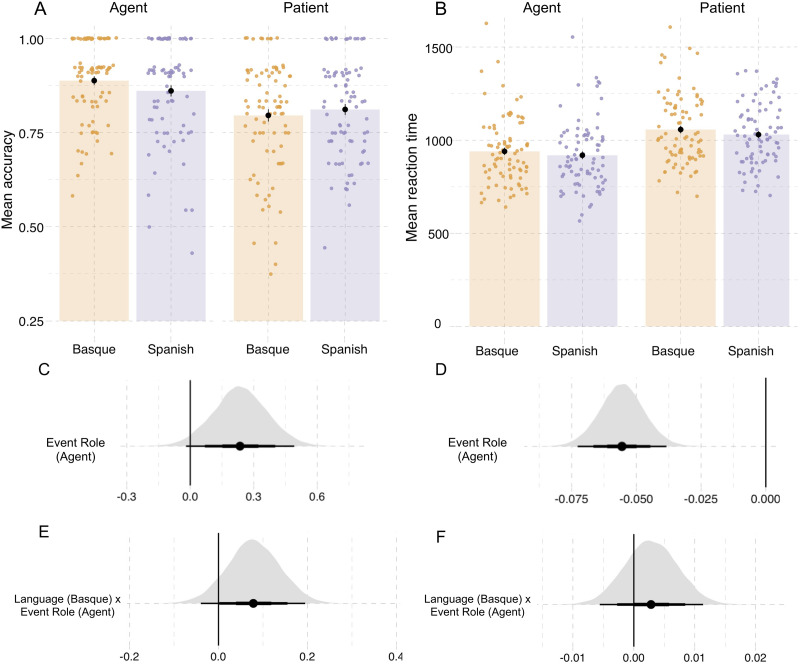
**Results from probe recognition task in Experiment 1.** (A) Recognition accuracy for probe pictures. (B) Reaction times (in milliseconds). Individual dots represent participant means, black dots represent the mean of participant means, and error bars indicate 1 standard error of the mean. Figure F2 in the Appendix shows fitted values from the Bayesian regression model. (C and D) Posterior estimates for the predictor event role from the Bayesian regression models for accuracy and reaction time, respectively (Tables D2 and D3). (E and F) Posterior estimates of the interaction between language and event role from the same models for accuracy and reaction time, respectively. Point intervals represent the 50%, 80%, and 95% credible intervals.

When reporting the parameter estimates of interest ($\hat{\beta }$), we provide the mean and standard error of the posterior draws. We additionally include the posterior probability of the hypothesis that the estimate is smaller than or larger than 0. This is equal to the proportion of draws from the posterior distribution that fall on the same side of 0 as the mean of the posterior distribution, which is a direct indication of the strength of the evidence (Kruschke, 2015). We visualize this information with posterior density plots for each parameter of interest (Figure 3B and Figure 4C–D).

### Results

The results from the event description task are shown in Figure 3 and from the probe recognition task in Figure 4; regression model summaries are presented in Tables D1, D2, and D3. Compared to patients, agents were described with greater specificity ($\hat{\beta }$_*Agent*_: mean = 1.13, SE = 0.15, P($\hat{\beta }$ > 0) = 0.99; see Table 1 and Figure 3). Participants also recognized agents with greater accuracy than patients in probe recognition ($\hat{\beta }$_*Agent*_: mean = 0.24, SE = 0.13, P($\hat{\beta }$ > 0) = 0.99; see Table 1 and Figure 4A).

**Table 1.** Proportion of specific or accurate responses in event description and probe recognition tasks, as well as reaction times (in ms) for probe recognition in Experiment 1; standard deviations in parentheses.

**Event Role**	**Language**	**Event description**	**Probe recognition**	
		**Proportion of specific responses**	**Proportion of correct responses**	**Reaction time**
Agent	Basque	0.90 (0.08)	0.89 (0.10)	939 (183)
Agent	Spanish	0.91 (0.09)	0.86 (0.13)	728 (113)
Patient	Basque	0.72 (0.09)	0.79 (0.14)	1057 (189)
Patient	Spanish	0.69 (0.10)	0.81 (0.13)	1030 (168)
Action	Basque	0.64 (0.11)	—	—
Action	Spanish	0.65 (0.12)	—	—

In line with the accuracy results, participants in both languages responded faster to agent probes compared to patient probes ($\hat{\beta }$_*EventRole*_ = −0.055, SE = 0.008, P($\hat{\beta }$ < 0) = 0.99; Table 1, Figure 4B).

The higher specificity, accuracy, and faster reaction times for agents were detectable despite the high variability between participants and events (for event description $\hat{\beta }$_*Participant*_: mean = 0.63, SE = 0.05, P($\hat{\beta }$ > 0) = 0.99; $\hat{\beta }$_*Event*_: mean = 0.91, SE = 0.10, P($\hat{\beta }$ > 0) = 0.99; for probe recognition: $\hat{\beta }$_*Participant*_: mean = 0.53, SE = 0.08, P($\hat{\beta }$ > 0) = 0.99; $\hat{\beta }$_*Event*_: mean = 0.69, SE = 0.10, P($\hat{\beta }$ > 0) = 0.99). This high variability was expected due to the online setting, which allows only limited control of participants’ behavior.

On top of these effects for event roles, we also found an interaction between language and event role in both tasks. In event description, Basque speakers described patients with higher specificity than Spanish speakers ($\hat{\beta }$_*Language*×*Patient*_ = 0.09, SE = 0.03, P($\hat{\beta }$ > 0) = 0.99; see Figure 3). By contrast, Spanish speakers described action verbs more precisely than Basque speakers ($\hat{\beta }$_*Language*×*Action*_ = −0.08, SE = 0.04, P($\hat{\beta }$ < 0) = 0.99). There were no notable differences in the specificity of agent descriptions between languages ($\hat{\beta }$_*Language*×*Agent*_ = −0.02, SE = 0.05, P($\hat{\beta }$ < 0) = 0.64).

In the probe recognition task (see Figure 4A; Table D2), the interaction between language and event role showed that the Basque participants were more accurate when responding to agent probes than the Spanish participants ($\hat{\beta }$_*Language*×*EventRole*_: mean = 0.08, SE = 0.06, P($\hat{\beta }$ > 0) = 0.91). In contrast, there was no substantial evidence for an interaction between language and event role in the reaction times ($\hat{\beta }$_*Language*×*EventRole*_ = 0.002, SE = 0.004, P($\hat{\beta }$ > 0) = 0.75; Table D3).

### Discussion

Speakers of Spanish and Basque both showed a general preference for agents in both tasks, reflected in higher specificity and accuracy for agents, as well as faster reaction times. This effect was consistent across languages and participants, even when the animacy of the patient was controlled for in the statistical analysis. This matches the results reported by Cohn and Paczynski (2013) and provides evidence for an agent preference in human cognition (New et al., 2007; Rissman & Majid, 2019). We will return to this finding in the General Discussion.

In addition to the general preference for agents over patients, there were differences between speakers of the two languages in their attention to event roles. In the probe recognition task, the Basque participants were more accurate than the Spanish participants in responding to agent probes, and less accurate than the Spanish participants in responding to patient probes. By contrast, we did not find any effect on the specificity of agent descriptions in the event description task. However, Basque speakers were more specific than Spanish speakers in describing patients.

Hence, we found effects of language on how speakers apprehended the event roles, although these were not consistent across tasks. A possible explanation for the divergent language effects between tasks could be the different time frames each task measured and the ability of the tasks to reflect event apprehension more or less directly (Firestone & Scholl, 2016). For event descriptions, the time required to type the responses could have led to memory decay (Gold et al., 2005; Hesse & Franz, 2010) and a deteriorated ability to reflect the apprehended information. Producing a sentence is a complex task and could also have contributed to memory distortion (Baddeley et al., 2011; Vandenbroucke et al., 2011). When producing descriptions, the words (lexical forms) of the agent and other parts of the sentence are usually retrieved in order of mention (Griffin & Bock, 2000; Meyer et al., 1998; Roeser et al., 2019). This potentially leaves the later elements of the sentence with a less clear memory trace. In Spanish, the patient is mentioned last (SVO order), while patients usually occupy the sentence-medial position in Basque (SOV order). This difference in word order could have interfered with the specificityof responses, because Basque speakers were able to “offload” patient information earlier (Baddeley et al., 2009, 2011). In fact, word order is known to influence the time course of sentence planning (Norcliffe et al., 2015; Nordlinger et al., 2022; Santesteban et al., 2015). In our experiment, all descriptions were agent-initial, and variations in word order were present only later in the sentence. Therefore, it does not appear likely that word order had an effect as early as in the apprehension phase, but we suggest that it affected how participants recalled and linearized the patient and action information when formulating their responses. It is also possible that additional post-hoc processes influenced the results because participants were not under time pressure to provide their answers.

By contrast, the time pressure was high in the probe recognition task because of the time-out. Participants responded by pressing the button on average 974 ms after the onset of the event picture (cf. Figure 2). This much shorter time between stimulus presentation and completed response may have reduced the effect of post-hoc cognitive processes, suggesting that the accuracy in this task better reflects the attention patterns during the event apprehension phase.

Therefore, the results from each task may reflect different stages of processing events: probe recognition accuracy would represent the outcome of event apprehension, and specificity in event descriptions would reflect a combination of event apprehension, possibly influenced by task demands and word order differences. These inherent differences between the tasks and the proneness of behavioral responses to be influenced by post-hoc processes make it necessary to further explore the event apprehension and to move beyond behavioral measures for doing so.

A way to bypass these problems and obtain a more direct measure of apprehension is to use eye tracking. Gaze allocation patterns to the briefly presented event pictures are considered direct reflexes of the event apprehension process (Gerwien & Flecken, 2016). When presented with an event picture (as in Figure 2), viewers collect parafoveal information on the event structure and use this coarse representation to decide where on the event picture to fixate first. Therefore, first fixations to event pictures can be reliably linked to the event apprehension process and provide an alternative to relying solely on offline measures. Measuring gaze allocation also provides a direct way of comparing the agent preference across tasks (in contrast to the behavioral task-specific measures in Experiment 1).

## EXPERIMENT 2

We adapted the design from Experiment 1 for the laboratory and introduced eye tracking to measure how participants directed their overt visual attention during event apprehension. Consequently, the main measure in Experiment 2 was the location of the first fixation in the stimulus pictures. We adapted the response modalities in the tasks to elicit faster responses, by requiring oral responses in the event description task and by reducing the time-out in the probe recognition task to 1500 ms. We predicted that the agent preference would be detectable in the fixation patterns and behavioral correlates, replicating and further characterizing the agent preference found in Experiment 1.

### Methods

#### Participants.

Native speakers of Basque (*N* = 38) and Spanish (*N* = 36) were recruited (age range = 18–40, mean age = 29, 49 female) and received monetary compensation for their participation. All Basque speakers and 29 Spanish speakers were tested in Arrasate (Basque Country); the other Spanish speakers participated in Zurich (Switzerland), due to constraints induced by the COVID-19 pandemic. In both locations, laboratories were set up *ad hoc* in school or university facilities and the same technical equipment was used. The experiment was approved by the ethics committees of the Faculty of Arts and Social Sciences of the University of Zurich (Approval Nr. 19.8.11) and the University of the Basque Country (Approval Nr. M10/2020/007). All participants gave written informed consent. All procedures are performed following the ethical standards of the 1964 Helsinki declaration and its later amendments.

#### Materials and procedure.

The stimuli were the same as in Experiment 1. The procedure followed mainly that of Experiment 1 but was adapted to the on-site setting and the eye tracking methodology. The experiment featured two consecutive blocks per task (instead of only one block per task in Experiment 1) with a self-timed pause between the tasks, i.e., after the second block. To characterize the two groups of participants (Basque speakers and Spanish speakers), individual differences measures were administered (see Table 2). Participants completed the Digit-Symbol Substitution Task from the Wechsler Adult Intelligence Scale (Wechsler, 1997) as a measure of perceptual and processing speed (Hoyer et al., 2004; Huettig & Janse, 2016; Salthouse, 2000). Participants also completed a lexical decision task to assess their lexical knowledge of Basque and Spanish (de Bruin et al., 2017) and completed a detailed questionnaire that collected information on their demographic profile, educational background, and linguistic habits.

**Table 2.** Language profiles and measures of linguistic competence and processing speed of participants in Experiment 2. Self-reported proficiency and lexical decision accuracy means could range from 1–10. The Digit Symbol Substitution Test scores ranged between 48–85. Standard deviations are given in parentheses.

**Measure**	**Basque group**	**Spanish group**
Self reported proficiency in Basque	9.08 (0.86)	3.72 (3.61)
Self reported proficiency in Spanish	7.64 (1.14)	9.65 (0.47)
Basque lexical decision task accuracy mean	8.97 (0.47)	5.88 (2.2)
Spanish lexical decision task accuracy mean	8.88 (0.75)	8.88 (0.83)
Digit Symbol Substitution Test results	68.5 (10.34)	64.5 (10.32)

#### Apparatus and data recording.

The experiment was programmed in E-Prime 2.0 (Schneider et al., 2002) and displayed on a 15.6″ computer screen with a resolution of 1920 × 1080 pixels. The participants placed their heads on a chin rest so that their eyes were at a distance of approximately 65 cm from the screen. The stimulus pictures subtended a visual angle of 11.73° horizontally (560 pixels) and 7.48° vertically (349 pixels). The center of each picture was 12.06° away from the central fixation cross on which the participants fixated at stimulus onset. Eye movements were recorded with a SMI RED250 mobile eye tracker (Sensomotoric Instruments, Teltow, Germany) sampling at 250 Hz. Button presses in the probe recognition task were recorded with a RB-844 response box (Cedrus, San Pedro, USA).

#### Data processing and analysis.

Data from six participants were lost due to technical errors or their inability to complete the experiment session. Twelve additional participants were excluded from the analysis due to the potentially heavy influence of the respective other language. This was determined to be the case when they reported using the respective other language frequently or preferentially (i.e., Spanish for native speakers of Basque or Basque for native speakers of Spanish), or when they scored equal or higher in the Basque lexical decision task than in the Spanish lexical decision task. The latter criterion was only applied to Spanish participants, because Basque speakers are usually very close or at the same level of performance as Spanish speakers due to diglossia. This exclusion criterion was applied to reduce the influence of possibly balanced bilinguals (Morales et al., 2015; Olguin et al., 2019; Yow & Li, 2015). The processing speed measures were similar in both language groups (cf. Table 2). On balance, 52 participants were included in the analyses (*N*_*Basque*_ = 28 Basque, *N*_*Spanish*_ = 24).

**Figure 5.**
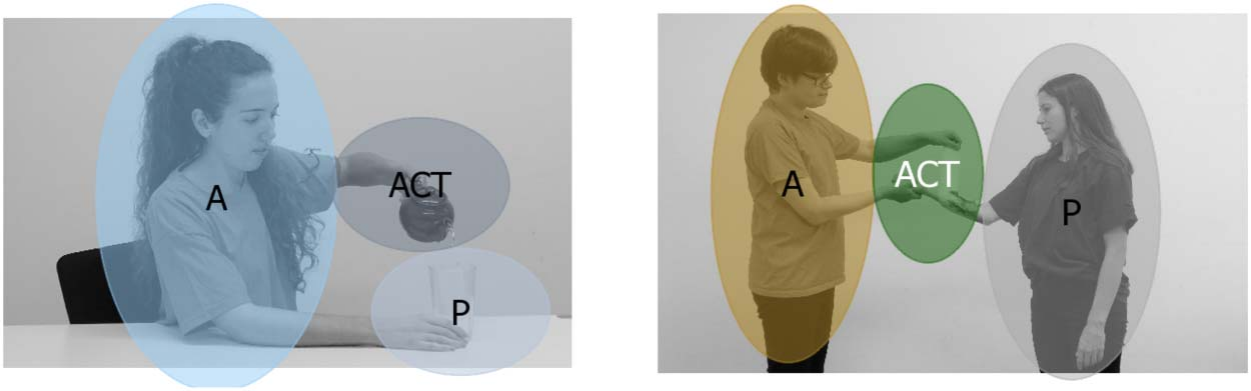
**Examples of agent (A), patient (P) and action (ACT) areas of interest for two stimuli.** Areas of interest were not visible to participants.

For each event picture, areas of interest for agents and patients were manually defined in the eye tracker manufacturer’s SMI BeGaze software (version 3.4). The areas of interest covered the face and upper part of the body for human characters and the whole object for inanimate patients (see Figure 5). We also defined an action area that encompassed the extended limbs of the agent (usually their hands) or the instruments involved in the action.^3^ The areas of interest for agents and patients were at least 30 pixels apart (mean = 74 pixels, *SD* = 38 pixels, corresponding to a visual angle of 1.85°) to avoid that fixations were assigned to the wrong area of interest due to measurement error.^4^ Fixations were detected using the algorithm implemented in SMI BeGaze.

For the eye tracking analyses, trials were excluded if participants’ fixations landed more than 100 pixels (visual angle of 2.5°) away from the edges of the target picture. Trials were also excluded if no response was given or, in the event description task, if the target verb and event roles were inverted (“Lisa heard Tim” instead of “Tim shouted to Lisa”). In total, 8821 trials were included in the first fixations analyses (93% of all trials).

The analysis of first fixations was the main measurement in Experiment 2, given that these fixations provide the most direct window into the visual event apprehension process (Gerwien & Flecken, 2016; Sauppe & Flecken, 2021). In addition, we also conducted an exploratory analysis of second fixations on the event pictures. Participants fixated first on the picture on average 192 ms (*SD* = 22 ms) after exposure. In some trials, participants subsequently launched another saccade, on average 318 ms after stimulus onset (*SD* = 29 ms), with a mean duration of 283 ms (*SD* = 129 ms). Because these saccades were launched when the brief exposure time was almost over, second fixations generally landed on the subsequently presented mask image. However, programming and executing a saccade takes between 100 and 200 ms (R. H. S. Carpenter & Williams, 1995; Duchowski, 2007; Pierce et al., 2019), which means that the second fixations were planned while the event picture was still visible, and often even during the execution of the first saccade, i.e., before the eyes landed on the picture for the first fixation. Thus, it seems likely that the second fixations are generated by similar mechanisms as the first fixations. This suggests that the second fixations, although not landing on the briefly exposed picture, may provide additional information on the event apprehension process (cf. Altmann, 2004; F. Ferreira et al., 2008, for additional discussion of the usefulness of “looking at nothing”). Analyses of second fixations were conducted on a subset of trials that exhibited second fixations that were directed at least 50 pixels away from the first fixation’s location. This distance threshold ensures that the second fixations represented genuine new fixations and not just measurement and classification errors. In sum, 4956 trials were analyzed for second fixations.

For specificity, accuracy, and response time analyses, the exclusion criteria and statistical modeling were identical to those in Experiment 1. In the event description analysis, 4663 trials were included (75% of all trials); in the probe recognition analyses, 2387 trials were included (95% of all trials).

As for Experiment 1, statistical analyses were conducted in R (R Core Team, 2022) using hierarchical Bayesian regression models through the brms (Bürkner, 2017, 2018) interface to Stan (B. Carpenter et al., 2017). Eye tracking analyses modeled the likelihood of fixating on the agent, patient, or action areas of interest. We fitted Bernoulli models for agent, patient, and action fixations separately. Models for fixations were fitted jointly for both tasks. Language, task, and their interaction were the predictors of interest. We included the following as nuisance predictors: the size of the target area of interest in pixels, the animacy of the patient, the task order, the closeness of the agent to the fixation cross, and the block order within each task. For the second fixations analyses, we also included the area of interest to which the first fixation was directed as a nuisance predictor to account for the correlation between fixation locations on such short time scales (Barr, 2008). The size of the areas of interest, the order of tasks, and the number of blocks were standardized (*z*-transformed) and categorical predictors were sum-coded (−1, 1). We included random intercepts and slopes for language and task by participant and item. We used Student-*t* distributed priors (*df* = 5, *μ* = 0, *σ* = 2) for the intercept and all population-level predictors. For random intercepts and slopes, we used a default prior (Student-*t*, 3 degrees of freedom, mean = 0, scale = 2.5). For accuracy and response time analyses, statistical models were specified like those in Experiment 1.

### Results

#### Fixations.

**Figure 6.**
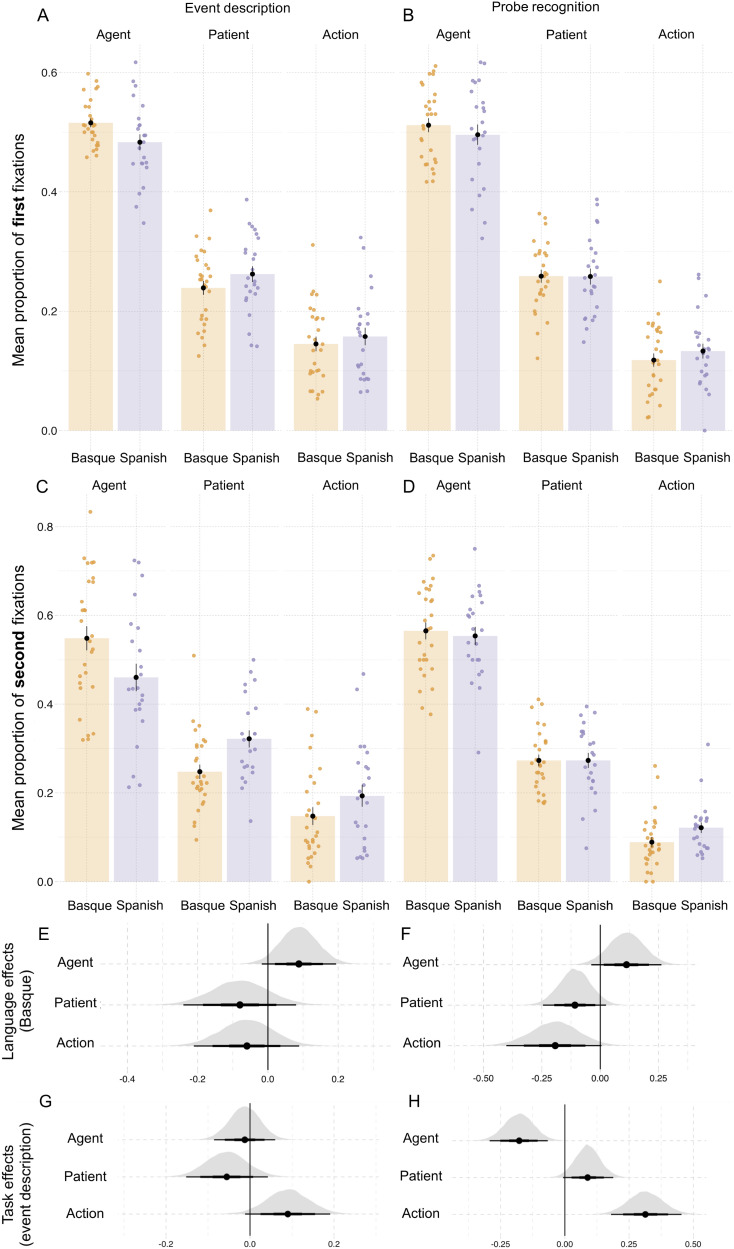
**Proportions of fixations to areas of interest in Experiment 2.** (A and B) First fixations. (C and D) Second fixations. Colored dots represent individual participant means and black dots represent the means of participant means; error bars indicate 1 standard error of the mean. (E and F) Posterior distributions of the estimates for the predictor language, for each of the three event roles (agent, patient, action, from separate models, cf. Tables D4, D5, D6) for first and second fixations, respectively. (G and H) Posterior distributions of the estimates for the predictor task, for each of the three event roles for first and second fixations, respectively. Point ranges represent the 50%, 80%, and 95% credible intervals. Corresponding plots showing fitted values from the regression models are shown in Figure F3.

**Figure E1.**
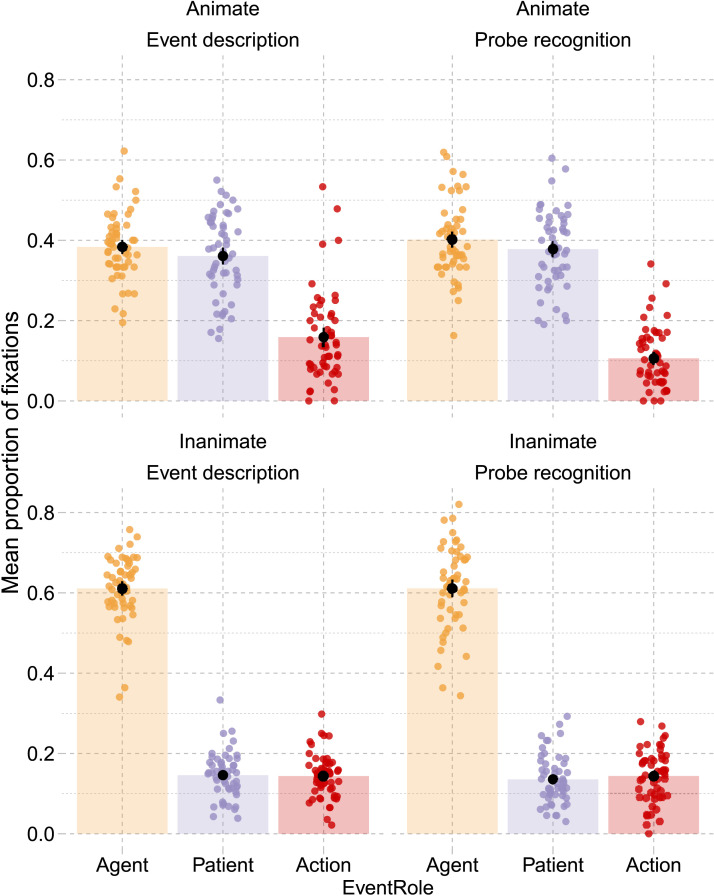
**First fixations to event roles in Experiment 2, grouped by task and animacy of the patient.** Animacy had a large impact on first fixations, but agents still received a higher number of fixations in the events where both agents and patients were human.

Overall, first fixations were directed primarily to agents (50.2% versus 25.2% to patients and 13.8% to actions). This effect was present in both language groups in both tasks (see Figure 6A–B). The animacy of the patient had a large effect on first fixations, but we still found more agent than patient fixations in the events where patients were animate (see Figure E1). For the second fixations, we again found a higher proportion of fixations to agents across language groups and tasks, although to a lesser degree than in first fixations (see Figure 6C–D).

Beyond a general preference to fixate on agents first, we also found effects of language and task on first fixations. The proportions of first fixations to the three areas of interest varied between the language groups (Figure 6A–B, Table 3). Basque speakers fixated more on agents than the Spanish speakers in both the event description and the probe recognition task ($\hat{\beta }$_*Language*_ = 0.09, SE = 0.05, P($\hat{\beta }$ > 0) = 0.96; Table D4). In turn, Spanish participants seemed to fixate more on patients ($\hat{\beta }$_*Language*_ = −0.08, SE = 0.08, P($\hat{\beta }$ < 0) = 0.84; Table D5) and actions ($\hat{\beta }$_*Language*_ = −0.07, SE = 0.08, P($\hat{\beta }$ < 0) = 0.81; Table D6), but these effects and the evidence for them were weaker.

**Table 3.** Proportions of fixations in Experiment 2 (means of participant means, standard deviations in parentheses).

**Event Role**	**Language**	**First fixations**	**Second fixations**
Agent	Basque	0.51 (0.03)	0.55 (0.10)
Agent	Spanish	0.49 (0.06)	0.51 (0.10)
Patient	Basque	0.24 (0.04)	0.25 (0.05)
Patient	Spanish	0.26 (0.05)	0.29 (0.07)
Action	Basque	0.13 (0.05)	0.11 (0.08)
Action	Spanish	0.14 (0.05)	0.16 (0.07)

For second fixations, language-related effects were similar to those found for the first fixations (Figure 6C–D), but more consistent across all event roles. Basque speakers looked more towards agents ($\hat{\beta }$_*Language*_ = 0.11, SE = 0.08, P($\hat{\beta }$ > 0) = 0.93; Table D4), while the Spanish speakers were more likely to fixate on the patient ($\hat{\beta }$_*Language*_ = −0.11, SE = 0.07, P($\hat{\beta }$ < 0) = 0.94; Table D5) and the action ($\hat{\beta }$_*Language*_ = −0.19, SE = 0.10, P($\hat{\beta }$ < 0) = 0.97; Table D6).

Fixation patterns were also affected by the task. Participants in both language groups launched more first fixations to the action ($\hat{\beta }$_*Task*_ = 0.10, SE = 0.05, P($\hat{\beta }$ < 0) = 0.97) and fewer fixations to patients ($\hat{\beta }$_*Task*_ = −0.06, SE = 005, P($\hat{\beta }$ > 0) = 0.89) in the event description task compared to probe recognition. In second fixations, participants in both languages were more likely to fixate on actions ($\hat{\beta }$_*Task*_ = 0.32, SE = 0.07, P($\hat{\beta }$ > 0) = 0.99) and less likely to fixate on agents ($\hat{\beta }$_*Task*_ = −0.18, SE = 0.06, P($\hat{\beta }$ < 0) = 0.99) in the event description task compared to probe recognition.

We did not find that language effects substantially differed by task in first fixations for any of the event roles ($\hat{\beta }$_*Language*×*Task*_ < 0.02, P($\hat{\beta }$ < 0) < 0.76 in all models). In contrast, language effects in second fixations more clearly varied by task: language differences were present in the event description task but not in the probe recognition task for agent fixations ($\hat{\beta }$_*Language*×*Task*_ = 0.07, SE = 0.06, P($\hat{\beta }$ > 0) = 0.89) and patient fixations ($\hat{\beta }$_*Language*×*Task*_ = −0.08, SE = 0.05, P($\hat{\beta }$ < 0) = 0.97). We did not find sufficient evidence for an interaction between task and language for action fixations ($\hat{\beta }$_*Language*×*Task*_ = 0.04, SE = 0.06, P($\hat{\beta }$ < 0) = 0.72).

**Figure G1.**
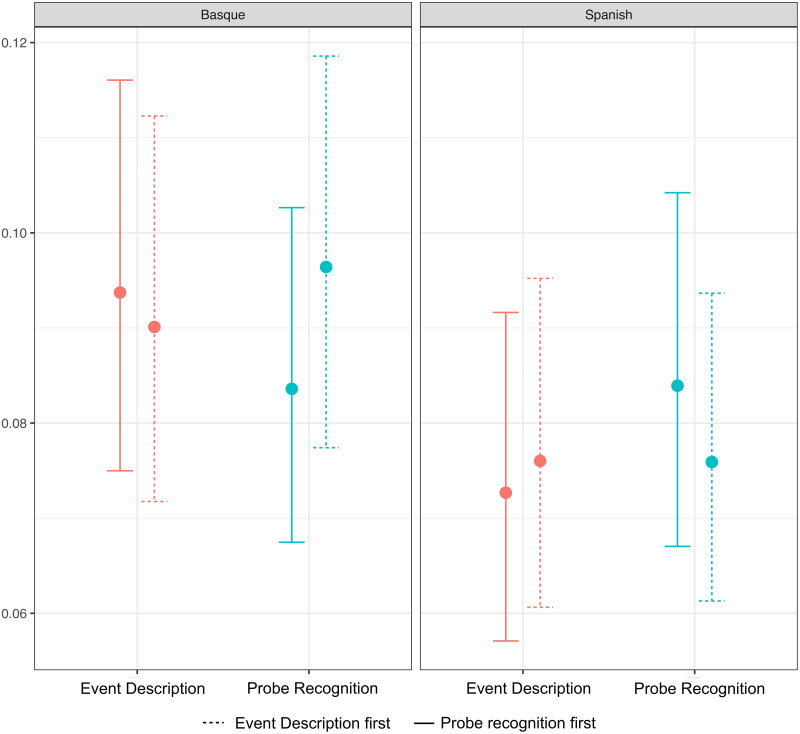
Conditional effects from the supplementary model for first fixations to agents with three way interaction between Language, Task and Task Order.

Additionally, we ran a supplementary analysis of first and second fixations to check whether the order of tasks (having event description task first or second) affected fixation patterns. For that, we fitted a model including a three way interaction between language, task and task order, keeping the rest of the model structure the same as in previous models. For first fixations to agents, we found evidence that task-order affected fixations ($\hat{\beta }$_*Language*×*Task*×*Task*−*Order*_ = 0.09, SE = 0.06, P($\hat{\beta }$ > 0) = 0.92); in Basque, there were more fixations to agents during probe recognition when it was the second task. In contrast, there was no such effect for Spanish speakers, i.e., fixations to agents were not affected in probe recognition when this task followed the event description task (see Figure G1 in Appendix G). For first fixations to actions, we found some evidence for an effect of task order in the opposite direction ($\hat{\beta }$_*Language*×*Task*×*Task*−*Order*_ = −0.08, SE = 0.08, P($\hat{\beta }$ < 0) = 0.84). This suggests that Basque speakers fixated more in the action area in probe recognition when this task was first, with no such effect of task order in Spanish. However, the evidence for this effect was rather weak. We did not find evidence that task order affected first fixations to patients ($\hat{\beta }$_*Language*×*Task*×*Task*−*Order*_ = −0.02, SE = 0.09, P($\hat{\beta }$ > 0) = 0.58). Similarly, we did not find evidence that task-order affected second fixations for any of the event roles (P($\hat{\beta }$ > 0) ≤ 0.80 for all models).

#### Specificity, accuracy and reaction times.

In both tasks, participants were overall more specific and accurate when describing agents or responding to agent probes compared to patients (event description specificity, $\hat{\beta }$_*Agent*_: mean = 0.89, SE = 0.16, P($\hat{\beta }$ > 0) = 0.99; probe recognition accuracy, $\hat{\beta }$_*Agent*_: mean = 0.16, SE = 0.12, P($\hat{\beta }$ > 0) = 0.90). Participants were also faster overall in responding to agent probes, compared to patient probes ($\hat{\beta }$_*Agent*_: mean = −0.04, SE = 0.01, P($\hat{\beta }$ < 0) = 0.99; see Figure 8, Table 4).

**Table 4.** Proportion of specific or accurate responses in event description and probe recognition tasks in Experiment 2; standard deviations in parentheses.

**Event Role**	**Language**	**Event description**	**Probe recognition**	
		**Proportion of specific responses**	**Proportion of correct responses**	**Reaction time**
Agent	Basque	0.92 (0.06)	0.91 (0.06)	701 (98)
Agent	Spanish	0.88 (0.08)	0.84 (0.14)	728 (113)
Patient	Basque	0.74 (0.09)	0.84 (0.09)	792 (109)
Patient	Spanish	0.71 (0.09)	0.82 (0.09)	765 (112)
Action	Basque	0.66 (0.09)		
Action	Spanish	0.68 (0.10)		

**Figure 7.**
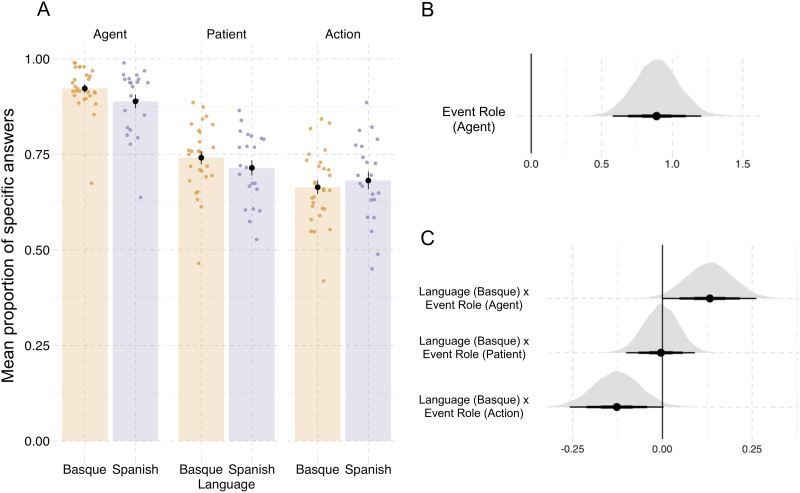
**(A) Proportion of *Specific* answers (in contrast to *General* or *Inaccurate* answers) in event description by language and event role in Experiment 2.** Individual dots represent participant means. Black dots represent means of participant means; error bars in indicate 1 standard error of the mean. (B) Posterior estimates for the predictor event role from the Bayesian regression model (Table D7). (C) Posterior estimates of interactions between each of the event roles (agent, patient, action) and language from the same Bayesian regression model. The three levels in the thickness of the point intervals represent the 50%, 80% and 95% credible intervals. Equivalent figures plotting fitted values are included in the Appendix, Figure F4.

We additionally found interactions between event roles and language in behavioral measures. In event description (Figure 7, Table 4), Basque speakers were more specific than Spanish speakers when describing agents ($\hat{\beta }$_*Language*×*Agent*_ = 0.13, SE = 0.07, P($\hat{\beta }$ > 0) = 0.98; Table D7), while less specific when describing actions ($\hat{\beta }$_*Language*×*Action*_ = −0.13, SE = 0.07, P($\hat{\beta }$ < 0) = 0.97). We did not find differences in the specificity of the patient descriptions ($\hat{\beta }$_*Language*×*Patient*_ = 0.00, SE = 0.04, P($\hat{\beta }$ < 0) = 0.53).

**Figure 8.**
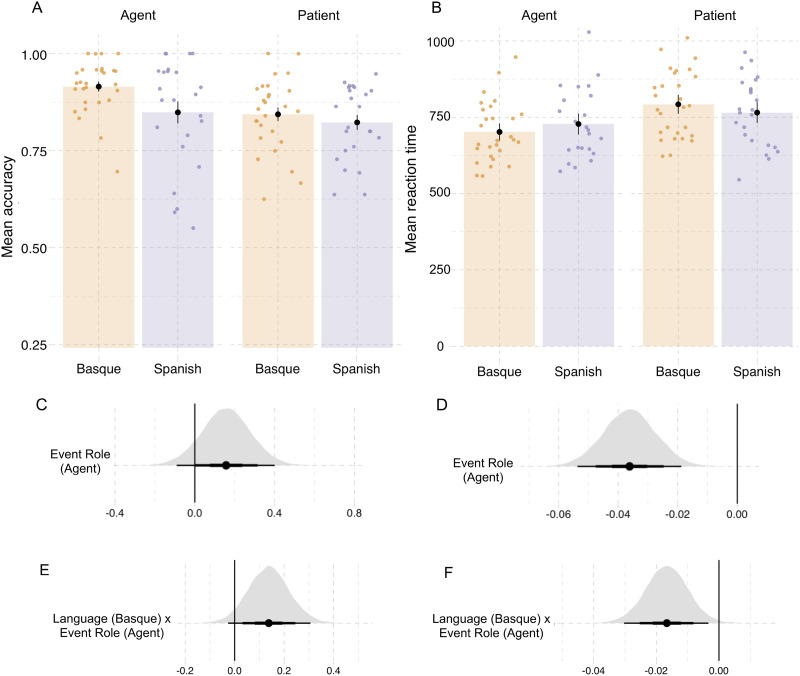
**Results from the probe recognition task in Experiment 2.** (A) Accuracy of probe detection. (B) Reaction times (button presses) in milliseconds. Individual dots represent participant means and black dots show means of participant means. (C and D) Posterior estimates for the predictor event role from the Bayesian regression models for accuracy and reaction time, respectively (Tables D8 and D9). (E and F) Posterior estimates of the interaction between language and event role from the same models for accuracy and reaction time, respectively. Point intervals represent the 50%, 80%, and 95% credible intervals. Equivalent figures plotting fitted values are shown in Figure F5.

In probe recognition (Figure 8, Table 4), Basque participants were overall more accurate than Spanish participants ($\hat{\beta }$_*Language*_ = 0.19, SE = 0.12, P($\hat{\beta }$ > 0) = 0.94; Table D8). Furthermore, the Basque participants were more accurate than Spanish participants in responding to agent probes compared to patient probes ($\hat{\beta }$_*Language*×*EventRole*_: mean = 0.14, SE = 0.08, P($\hat{\beta }$ > 0) = 0.95).

This correlated with the effects in reaction times. Basque speakers were on average 27 ms faster responding to agent trials and 26 ms slower responding to patient trials than participants in the Spanish group ($\hat{\beta }$_*Language*×*EventRole*_: mean = −0.016, SE = 0.006, P($\hat{\beta }$ < 0) = 0.99, Figure 8B, Tables 4 and D9).

### Discussion

The preference for agents observed in behavioral measures in Experiment 1 was replicated in Experiment 2. Fixation data provided additional evidence and expanded it to a measure that more closely reflects event apprehension processes. Eye movements (and especially first fixations) are a direct measure of the reflexes of event apprehension and are therefore assumed not to be as susceptible to post-hoc processes as the other behavioral measures. Hence, our results point to an agent preference in the earliest stage of event processing, across both languages and tasks tested.

Experiment 2 additionally provided evidence that language and task demands can modulate attention to event roles. Effects were small in size, but seemed to show that Basque speakers devoted more overt visual attention to agents than Spanish speakers, and, in turn, that Spanish speakers inspected patients and actions more often than Basque speakers. The effects were clearest in the measurements for the agent role. We also found a trend for patients and actions, although the effects for these roles were less strong and clear. The presence of effects between language groups suggests that the case marking of agents in language can modulate visual attention during event apprehension.

As for task effects, fixation data in Experiment 2 allowed a direct comparison of the effect of the upcoming task on event apprehension (unlike in Experiment 1, which relied solely on task-specific behavioral measures). Task effects showed that participants inspected action areas more in the probe recognition than in event descriptions, while the opposite was true for agents.

In fact, the gaze allocation patterns in Experiment 2 seem to provide detailed information on the subprocesses underlying event apprehension and how task demands would affect them. The fixation distributions suggest that there is an initial phase of apprehending the depicted event that is less susceptible to task demands. This is followed by a second phase that is more malleable and more strategically tuned to the task. When presented with the picture, the participants had access to coarse-grained, parafoveally perceived information to launch their first fixation. The coarse-grained nature of the information available before fixating on the picture may have limited the influence of task-specific demands for choosing the specific fixation location. By contrast, to decide on the location of the second fixation, participants benefited from a closer view of the event. This means that they had the opportunity to fine-tune their gaze to task-relevant areas. Indeed, the second fixations showed larger effects of task (and also language-task interactions). Participants in both language groups made more second fixations on action areas in the event description task than in the probe recognition task. This was most likely because the action information was crucial for the description task but not necessary for the recognition task. This suggests that participants were able to monitor their gaze more strategically and adapt to the task demands for the second fixations.

Thus, it is likely that the second fixations reflect a more flexible and refined process compared to the first fixations, with an increased influence of top-down factors such as the task and language requirements. A first fixation might suffice to extract coarse event-level information, including general information about the identity of event roles (cf. Dobel et al., 2011; Flecken et al., 2015). A second fixation, in turn, serves to further inspect the most relevant event areas in a strategic fashion.

An open question in this context is the benefit of launching the second fixations given their timing. In the majority of the trials, the brief exposure picture had already disappeared by the time this fixation landed on its target so that participants did not profit from additional foveally obtained visual information. Nonetheless, directing the gaze towards an “empty” area might have helped participants retrieve valuable information stored in their visual working memory about the event element previously located in that space (Altmann, 2004; F. Ferreira et al., 2008; Staudte & Altmann, 2017). Second fixations could thus reflect a memory retrieval process aimed at maximizing the visual representation of the event to perform more accurately in the task. Alternatively, second fixations might have been planned in the hope of still obtaining more visual input from the picture, as these were often planned during the execution of the first saccade.

## GENERAL DISCUSSION

Our two experiments show evidence of an agent preference in visual attention across tasks and languages. This is reflected in early fixations to event participants, as well as in accuracy and reaction times. This indicates that event apprehension is driven by a deeply rooted cognitive bias to inspect agents in the first place. In addition to an agent preference, we also found that language-specific and general task demands modulated overt visual attention allocation. Basque speakers directed slightly more attention to agents than Spanish speakers in the event apprehension process. Overall, there were fewer fixations to agents and more fixations to actions in the event description task than in the probe recognition task. Together, these findings suggest that the agent preference is an early and robust mechanism in event cognition and that it interacts with other top-down mechanisms. In the following sections, we discuss these findings and their implications.

### The Agent Preference as a Cognitive Universal

Speakers of Basque and Spanish allocated more attention to and were more accurate with agents than with patients or actions. This effect was robust and consistent across all measures in both experiments. Fixation data showed that this preference already emerged in the first fixations. The decision for the first saccade is made within the first 50–100 ms after the event picture is available and thus before the influence of any potential post-hoc reasoning. This early timing indicates that the preference for agents is spontaneous, as expected if it is a general cognitive bias.

The preference for agents also persisted when the patients were human, demonstrating that animacy (or humanness) was not the only factor driving this effect. Previous studies have used events that involved inanimate and smaller-sized patients, in events such as a woman cutting a potato (Gerwien & Flecken, 2016; Sauppe & Flecken, 2021). Character size (Wolfe & Horowitz, 2017) and animate motion (Pratt et al., 2010) are known to drive visual search and probably biased visual attention towards the agents in these previous studies. In the current study, we included larger-sized inanimate patients (e.g., a large plant) and animate patients (e.g., in kicking or greeting events), and still found a preference for agents. Our findings thus show that the agent preference is critically sensitive to event role information, and not animacy or salience alone.

The preference for agents was also consistent across tasks. In the event description task, this was expected because participants had to attend to the event to produce a written or oral description. Given that the canonical word order of Basque and Spanish is agent-initial, it is likely that the early attention to agents was driven by sentence planning demands. In picture description studies, agents are fixated more during early planning for the preparation of agent-initial sentences compared to patient-initial sentences (Griffin & Bock, 2000; Norcliffe et al., 2015; Nordlinger et al., 2022; Sauppe, 2017). Early agent fixations in the event description task are thus in line with what Slobin (1987) termed “thinking for speaking” because they constitute a preparatory step for sentence production.

However, the results of the probe recognition task also showed a preference for agents. This was the case even though the participants were not required to produce any linguistic output and could solve the task (answering by a button press whether a person or object had appeared in the previously shown target picture) without employing language. Thus, the preference for agents when visually scanning the picture in this task cannot be explained by the preparatory demands of sentence planning. The probe pictures were balanced with respect to whether they showed the agent or the patient, so neither event role was privileged in the experimental design. The finding that agents were still preferred thus points to a general top-down bias to search for agents, independent of task demands.

This result is consistent with the findings in Cohn and Paczynski (2013), who presented participants with depictions of agents and patients in cartoon strips. They measured looking times to each role while participants performed a non-linguistic task (rating how easy the event was to understand). Cohn and Paczynski found longer viewing times for agents than for patients and interpreted this as evidence that agents were initiating the building of event structures.

By contrast, our results are inconsistent with the findings by Hafri et al. (2018), who asked participants to indicate by button press whether a target actor appeared on the right or left side. This elicited longer response times for agents than for patients. In our study, however, response times were shorter for agents than for patients. The difference in the results patterns could be related to the level of role encoding encouraged by each task. In Hafri et al.’s study, the target actor that the participants had to identify was defined by a low-level feature (the gender or color of clothing). Instead, our probe recognition task required participants to recognize whether a character or object was present in the previously presented event. This means that participants could not only rely on low-level visual features but had to encode character information as a whole to answer the task, especially in events with human agents and human patients. This may have required participants to pay more attention to the event roles and their relations. Nonetheless, our results and the results from Hafri et al. (2018) converge in showing that event roles are detected and processed spontaneously, even when this is not necessary for the task.

On another level, agency is likely composed of multiple factors or cues (such as animacy or body posture) that each attract attention individually (Cohn & Paczynski, 2013; Gervais et al., 2010; Hafri et al., 2013; Verfaillie & Daems, 1996). The prominence of agents could also be related to their role as the initiator of the action. Cohn and Paczynski (2013) argue that agents initiate the building of event representations and provide anticipatory information on the rest of the event. Similarly, it has been proposed that agents are at the head of the causal chain that affects patients (cf. also Dowty, 1991; Kemmerer, 2012; Langacker, 2008). Active body postures are also known to attract attention and cue the processing of agents (Gervais et al., 2010). Our findings do not provide information on these individual mechanisms, but do show that agents on the whole are preferentially attended to already at the apprehension stage. Future research should test the degree to which the agent preference is based on each of its underlying factors.

The preference for agents in our experiments is, furthermore, consistent with the findings of an agent preference in grammar (Dryer, 2013; Fillmore, 1968; Napoli & Sutton-Spence, 2014; V. A. D. Wilson et al., 2022) and sentence comprehension (Bickel et al., 2015; Demiral et al., 2008; Haupt et al., 2008; Wang et al., 2009). V. A. D. Wilson et al. (2022) propose that grammar arises from general principles of event cognition. Based on evidence from primates and other species, V. A. D. Wilson et al. suggest that agent-based event representations are phylogenetically old and were already present in the ancestors of modern humans. From this it follows that an agent preference should be a likely candidate for a genuinely universal trait in human cognition (Henrich et al., 2010; Rad et al., 2018; Spelke & Kinzler, 2007). While our results are consistent with this, future studies should expand the sample and explore whether the agent preference not only generalizes across different languages but also across different cultural and social traditions (Henrich et al., 2010). This is crucial to ensure that this preference exists in the diversity of human populations and is not the by-product of Western education or cultural practices. Future research should also test the domain generality of the agent preference, for example, in lower-level perceptual tasks, and probe its neurobiological underpinning, with regard to the time courses and spatial distributions of neural activity (Kemmerer, 2012).

### The Agent Preference as Modulated by General and Language-Specific Task Demands

In addition to a general preference for agents, our results also showed modulations of the attention to event roles following language and task differences. These effects were smaller and less consistent than the main effects for agents. Albeit weaker, this evidence is still valuable and seems to point to a more nuanced picture of the processes underlying visual attention in event apprehension, where the agent preference interacts with other top-down mechanisms.

On the level of general task effects, we found that fixations to actions increased and fixations to agents decreased in event description compared to probe recognition, in both Basque and Spanish. These differences in visual attention probably reflect the diverging affordances required by each task. In event description, participants needed to obtain information on the relationship between event roles to plan the verb and describe the event as a whole (Griffin & Bock, 2000; Norcliffe & Konopka, 2015; Sauppe, 2017). This probably biased participants to the action area in the event and attenuated the general agent preference. In contrast, the probe recognition task only required identifying one of the event roles, and the relationship between the agent and the patient was irrelevant to the task. This likely explains the increased attention to agents in this task. Hence, general task demands exert a top-down influence on event apprehension. Knowing what kind of information is required to perform a task allows viewers to adapt the uptake of visual information. This supports the idea that event perception is guided by prior expectations and context (Gilbert & Li, 2013; Henderson et al., 2007).

Producing language is also a task demand, and it impacted how participants inspected events during apprehension. Basque speakers paid more overt visual attention to agents than Spanish speakers. A general tendency to first look at agents was also observedamong Spanish speakers, but to a lesser extent than among Basque speakers. The language effects were small, although the size of the effects is within the range observed in previous brief exposure studies (Gerwien & Flecken, 2016; Hafri et al., 2018; Sauppe & Flecken, 2021).

Language effects were consistent across fixations and the behavioral measures in Experiment 2. Although event apprehension has been argued to be a prelinguistic process (i.e., to take place before language-related processes start, Griffin & Bock, 2000), our evidence seems to show that it is impacted by grammatical differences between languages.

When describing event pictures, Basque speakers need to decide early on which role the subject has, presumably based on information about agency in the picture (such as body posture). Obtaining information about agency would be necessary to commit to a sentence structure and to prepare the first noun phrase, i.e., to decide whether to use the ergative case or not at the beginning of the planning process (Griffin & Bock, 2000; Norcliffe & Konopka, 2015). In comparison, Spanish speakers could plan the subject noun phrase without committing to whether it is an agent or not because the form of the noun phrase remains the same in either situation. This might be especially so when time pressure is high and speakers plan sentences highly incrementally, i.e., in small units (F. Ferreira & Swets, 2002). Spanish allows greater flexibility in incremental sentence planning (at least for this particular grammatical feature), and so speakers of this language can defer their decision on which sentence structure to produce and the semantic role of the first noun phrase (Norcliffe et al., 2015; Stallings et al., 1998). This may allow Spanish speakers to attend more to other aspects of events during early visual inspection (van de Velde et al., 2014; Wagner et al., 2010).

This might explain the higher proportion of fixations to agents in Basque than in Spanish. This interpretation is consistent with previous picture description studies with ergative-marked sentences. In eye tracked picture description studies on Hindi and Basque, Sauppe et al. (2021) and Egurtzegi et al. (2022) showed that speakers looked more towards agent referents in the relational-structural encoding phase (approximately the first 800 ms) of sentence planning when preparing sentences with unmarked agents as first noun phrases. In comparison, when planning sentences with ergative-marked agent noun phrases, speakers distributed their visual attention more between agents and the rest of the scene. Sauppe et al. and Egurtzegi et al. propose that this difference in gaze behavior arises from speakers’ need to commit to the agentivity and thereby the case marking of first noun phrases earlier when planning ergative marked sentences. In our experiments, participants would obtain rough information on event roles from parafoveal vision, and they would be guided by the agent preference for launching their first fixation. In this process, Basque speakers would be influenced even more by thiscognitive bias, given their sentence planning demands (Egurtzegi et al., 2022). Hence, language demands would exert a pressure and modulate the attention to events via rapid cognition.

However, the effects of language on the attention to event roles need to be considered cautiously in the current experiments for several reasons. On the one hand, the behavioral results in the event description task in Experiment 1 yielded a different pattern of language effects than those in Experiment 2: we found higher agent specificity for Basque speakers in Experiment 2, but not in Experiment 1. Although this might be due to the changes introduced in the task modality (written vs. oral responses), it is not clear whether this can fully explain the divergence in language effects. In addition, the language manipulation we employed was between groups. Hence, it could be that the differences between Basque and Spanish speakers in our experiments were caused by cultural, educational, or other differences between the groups. In our experiments, the participants came from the same general population and their educational level was also largely similar. This reduces the possibility that group differences caused the effect; however, only a within-group manipulation with highly proficient, fully balanced bilingual participants could rule it out.

Finally, the probe recognition task in the studies reported here also showed differences between Basque and Spanish speakers in their attention to event roles. The effects were in the same direction as the ones in the event description task and were also reflected in first fixations and behavioral measures. Given that this task did not require overt linguistic output, there are several possibilities for the source of the differences between Basque and Spanish. Event apprehension might be affected by language even when performing tasks that do not explicitly require overt linguistic responses. This could happen either through a long-lasting impact of linguistic experience or be caused by the covert recruitment of language in the probe recognition task. The interaction we found between task order and language effects possibly indicates that there were carry-over effects of language from the event description to probe recognition task, and this could support the account of covert recruitment of language. Another way for future research to investigate the origin of language effects would be to replicate the current study in the progressive aspect, where the ergative mark is not used in Basque. In this setup, the planning demands would be the same across language groups, and hence any differences between language groups would be related to long-lasting effects of language.

Other grammatical differences between languages have also been found to modulate attention to events, both in linguistic and non-linguistic tasks (e.g., Athanasopoulos & Bylund, 2013). Flecken et al. (2015), for example, found different neural responses for English and German speakers when they were presented with a picture that did not match a previous event sequence, and differences between speakers were correlated with language-specific characteristics. Consistent with this literature, our experiments provide tentative evidence that language might modulate attention in the event apprehension stage. However, future studies with within-group manipulations should clarify whether and how language can modulate attention to events under different task demands.

## CONCLUSIONS

The current behavioral and eye tracking data show evidence of a general tendency to attend to agents during the processing of events from early on, already at the event apprehension phase, also for events with animate patients. This supports the hypothesis that there is a general preference for agents in human cognition (Rissman & Majid, 2019; V. A. D. Wilson et al., 2022). We additionally find that this agent preference persists across two typologically different languages as well as across two task modalities. Our findings are therefore consistent with evidence that event role hierarchies are similar across different languages (Ünal, Ji, & Papafragou, 2021; Ünal, Richards, et al., 2021), despite substantial variation in grammatical encoding (Bickel, 2010; Bickel & Nichols, 2009).

In the current study, the attention to event roles also followed language and task differences. The evidence for these effects suggests that general and language-specific task demands can modulate (but not override) the way speakers visually inspect agent and patient roles. Future studies investigating universal event role processing mechanisms (Dobel et al., 2007; Hafri et al., 2018) should expand the empirical basis by including more diverse stimuli and task demands, and more diverse languages (Blasi et al., 2022; Henrich et al., 2010; Majid & Levinson, 2010).

## ACKNOWLEDGMENTS

Balthasar Bickel and Sebastian Sauppe share the senior authorship of this article. We thank Alexandra Bosshard, Noelia Falcón García, Ruben Ruben Mögel, and André Müller for help with stimulus creation; Giuachin Kreiliger for support with statistical analyses, and Oskar Elizburu and Alfabetatze Euskalduntze Koordinakundea for providing access to a laboratory space. We also thank Ted Gibson, Alon Hafri, and two anonymous reviewers for valuable comments on an earlier version of this paper.

## AUTHOR CONTRIBUTIONS

Arrate Isasi-Isasmendi: Conceptualization; Data curation, Formal analysis; Investigation; Methodology; Resources; Software; Visualization; Writing – Original draft; Writing – Review & editing. Caroline Andrews: Conceptualization; Formal analysis; Methodology; Software; Supervision; Writing – Original draft; Writing – Review & editing. Monique Flecken: Conceptualization; Writing – Review & editing. Itziar Laka: Conceptualization; Resources; Writing – Review & editing. Moritz Daum: Conceptualization; Writing – Review & editing. Martin Meyer: Conceptualization; Funding acquisition. Balthasar Bickel: Conceptualization; Formal analysis; Funding acquisition; Resources; Supervision; Writing – Review & editing. Sebastian Sauppe: Conceptualization; Formal analysis; Funding acquisition; Methodology; Project administration; Supervision; Writing – Original draft; Writing – Review & editing.

## FUNDING INFORMATION

This work was funded by the Swiss National Science Foundation (SNSF project grant number 100015_182845, BB and MM) and the National Center for Competence in Research “Evolving Language” (SNSF agreement number 51NF40_180888, BB, MM, and MMD) and a travel grant from the Graduate Research Campus, University of Zurich (AII). IL was supported by a grant from the Basque Government (IT1439-22).

## DATA AVAILABILITY STATEMENT

Raw data, annotations of responses, analysis scripts, and more example stimuli can be found online at https://osf.io/c5ubv/.

## Notes

^1^ Abbreviations: ERG: ergative case; NOM: nominative/unmarked case; AUX: auxiliary verb, DET: determiner.^2^ In the present study, “Basque” and “Spanish” refer only to the language groups in this experiment, and do not aim to convey information about participants’ nationality or identity.^3^ For two events (“shout at” and “scare”) no action areas of interest were defined because it was not possible to locate an area in the picture that solely belonged to the action. These events were only analyzed for agent and patient areas.^4^ The eye tracker’s gaze position accuracy is given as 0.4° by the manufacturer.

## Figures and Tables

**Table D1.**
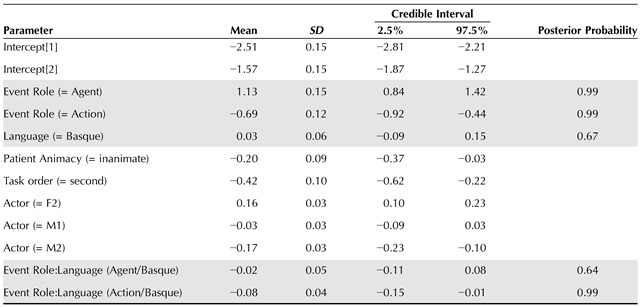
Results of the Bayesian cumulative regression modeling event description specificity in Experiment 1.

**Table D2.**
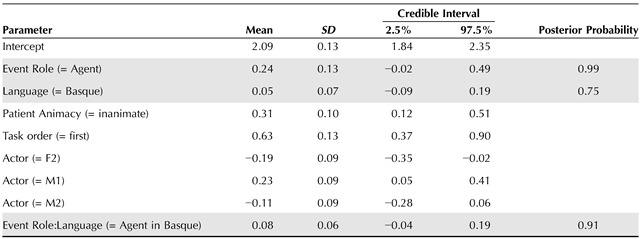
Results of the Bayesian Bernoulli regression modeling the likelihood of probe recognition accuracy in Experiment 1.

**Table D3.**
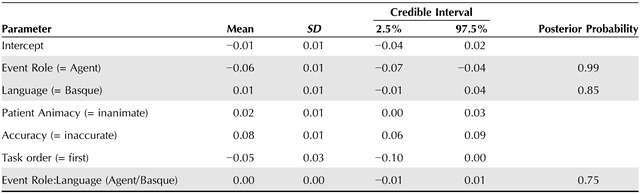
Results of the Bayesian Gaussian regression modeling the logarithm of button press reaction times in Experiment 1.

**Table D4.**
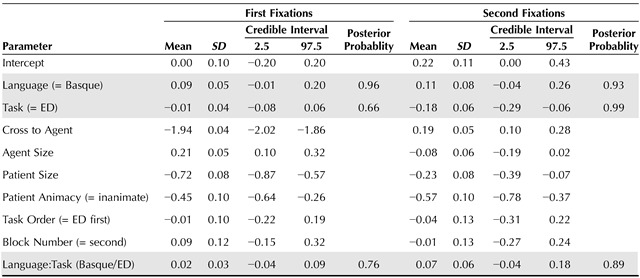
Results of the Bayesian Bernoulli regression modeling the likelihood of fixations on the agent area of interest in Experiment 2.

**Table D5.**
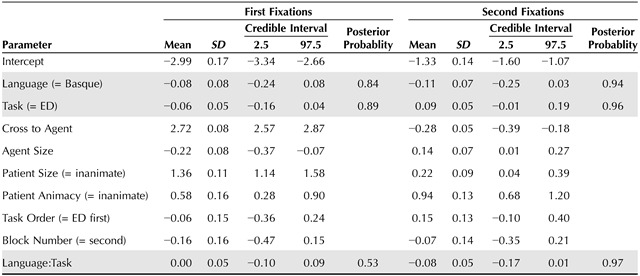
Results of the Bayesian Bernoulli regression modeling the likelihood of fixations on the patient area of interest in Experiment 2.

**Table D6.**
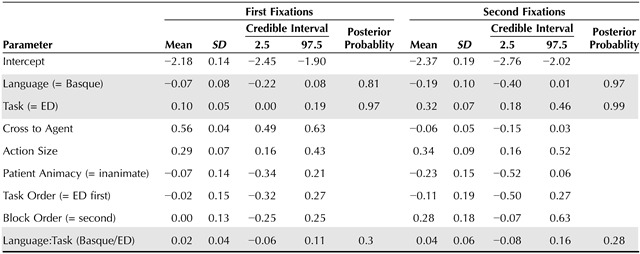
Results of the Bayesian Bernoulli regression modeling the likelihood of fixations on the action area of interest in Experiment 2.

**Table D7.**
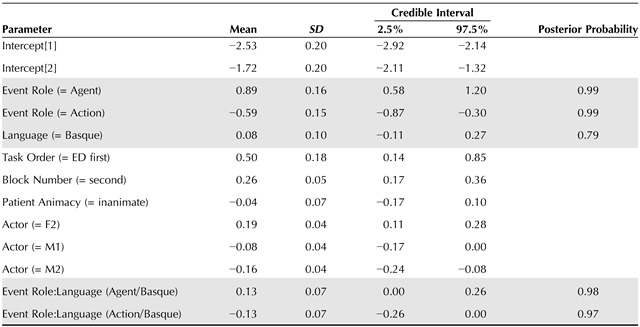
Results of the Bayesian cumulative regression modeling event description specificity in Experiment 2.

**Table D8.**
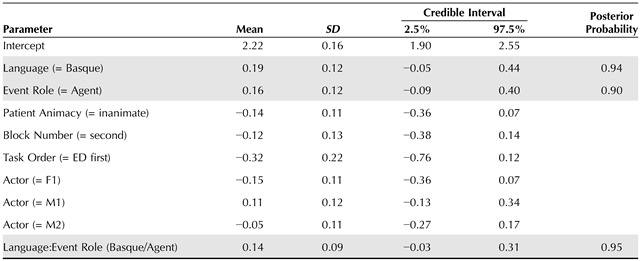
Results of the Bayesian Gaussian regression modeling the likelihood of probe recognition accuracy in Experiment 2.

**Table D9.**
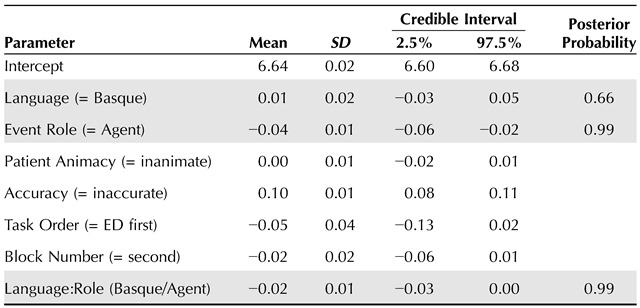
Results of the Bayesian Gaussian regression modeling the logarithm of button press reaction times in Experiment 2.

**Figure F1.**
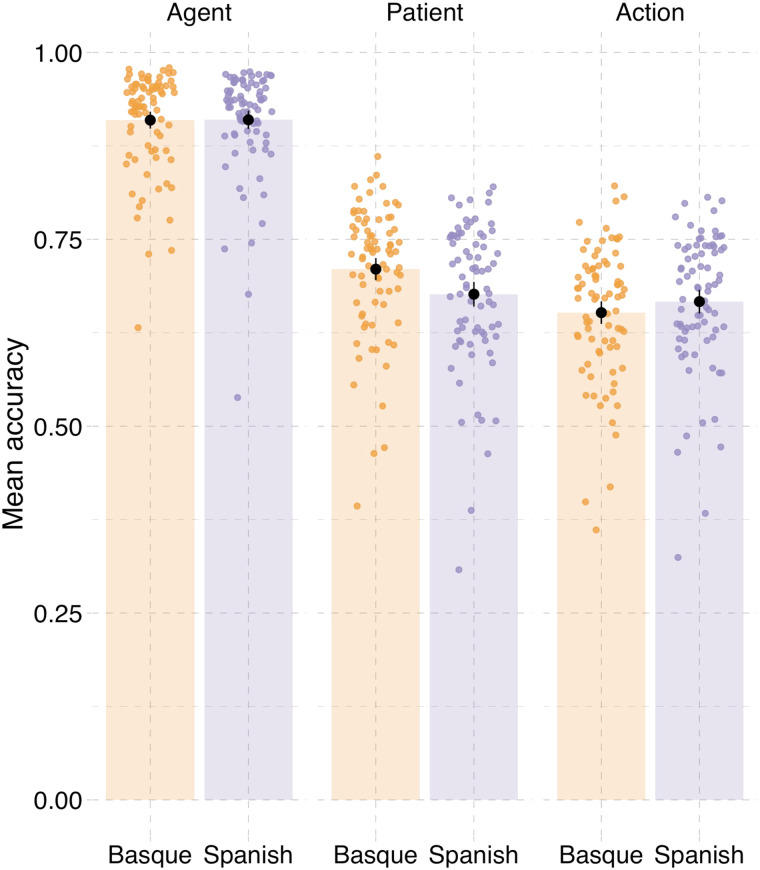
**Accuracy results in the event description task for Experiment 1, plotted from fitted values extracted from the Bayesian regression models and grouped by language and event role.** The equivalent figure with observed data is 3.

**Figure F2.**
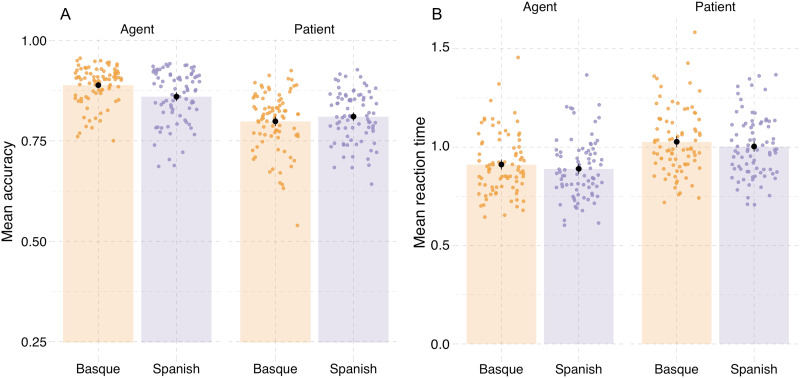
**Results in the probe recognition task for Experiment 1, plotted from fitted values extracted from the Bayesian regression models and grouped by language and event role.** (A) Accuracy results. (B) Reaction time results. The equivalent figure withobserved data is 4.

**Figure F3.**
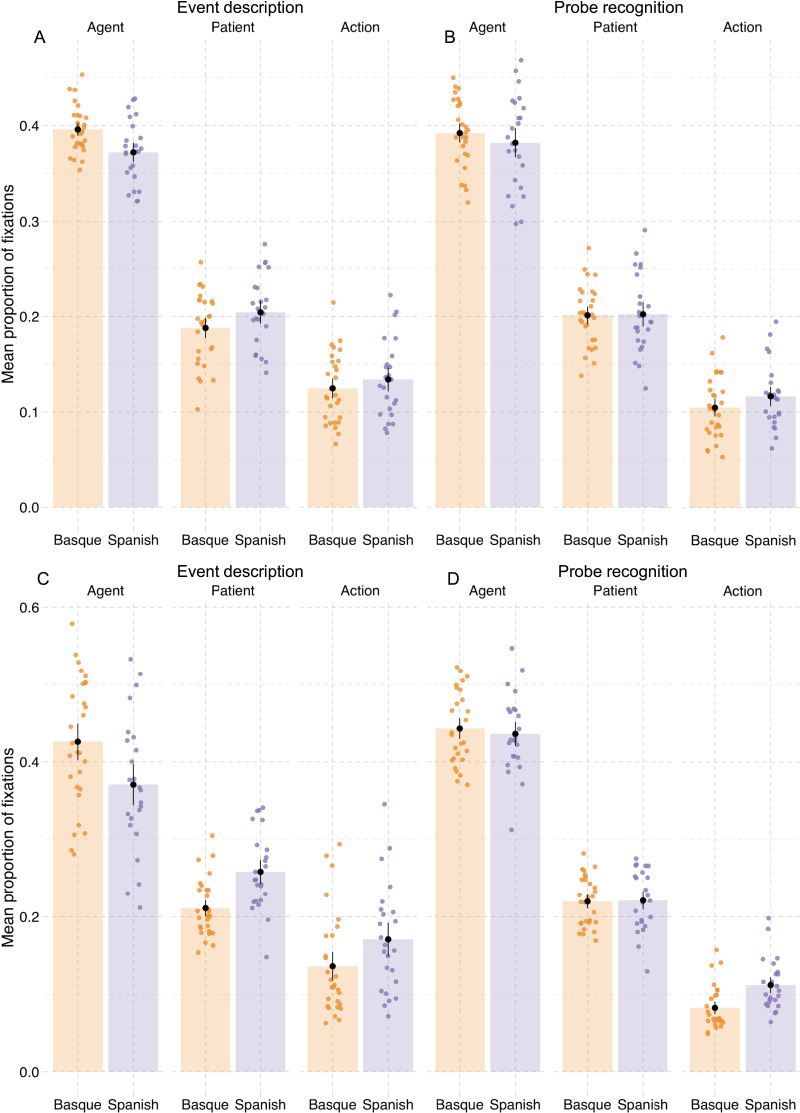
**Fixation results in the event description (left) and probe recognition (right) tasks, plotted from fitted values extracted from the Bayesian regression models and grouped by language and event role.** (A) First fixations. (B) Second fixations. The equivalent figure with observed data is 6.

**Figure F4.**
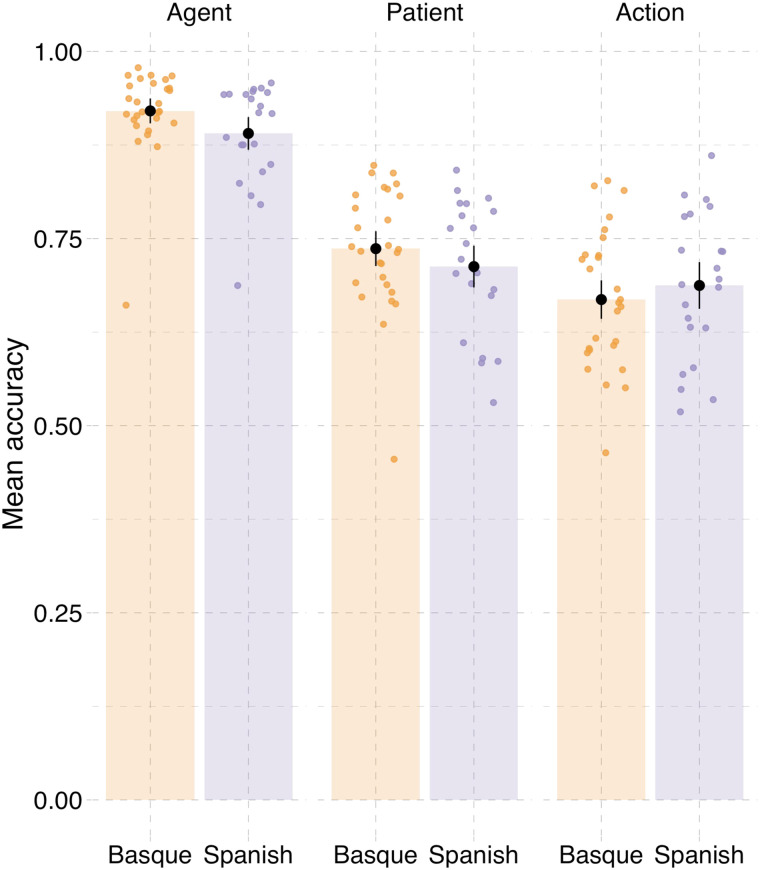
**Accuracy results in the event description task for Experiment 2, plotted from fitted values extracted from the Bayesian regression models and grouped by language and event role.** The equivalent figure with observed data is 7.

**Figure F5.**
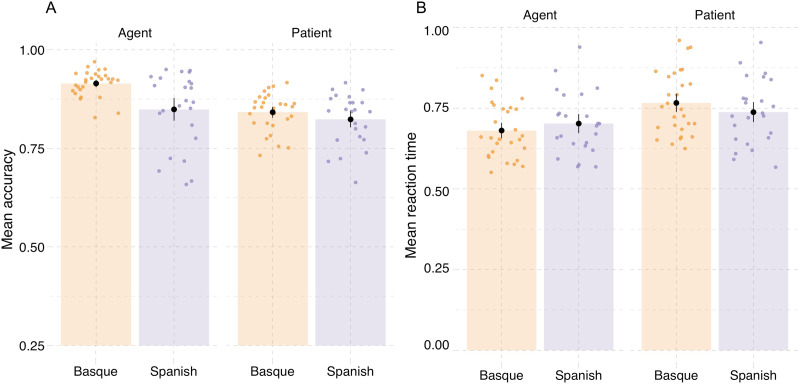
**Results in the probe recognition task for Experiment 2, plotted from fitted values extracted from the Bayesian regression model and grouped by language and event role.** (A) Accuracy results. (B) Reaction time results. The equivalent figure with observed data is 8.

## APPENDIX A: EVENTS DEPICTED IN THE STIMULI (*N* = 58)

**Table A1.** List of events depicted in experimental stimuli.

**Human Agent, Inanimate Patient**	**Human Agent, Human Patient**	**Intransitive**
Light candle	Poke	Lean
Crack egg	Grab	Yawn
Clean Blackboard	Scratch	Sleep
Read book	Strangle	Jump
Cut potato	Kick	Kneel
Trim plant	Pull	Sneeze
Peel mandarin	Push	Trip
Play drum	Greet	Sit down
Fix bike	Hit	Stretch
Put on glove	Tread on	Crouch
Open box	Drag	
Water plant	Help get up	
Lift table	Shout at	
Push shelf	Tie up	
Drag bag	Pinch ear	
Mix flour	Beckon	
Pick up basket	Brush
Pour water	Scold	
Tear paper	Tickle	
Open umbrella	Bandage	
Hang up laundry	Scare	
Wipe bowl	Feed	
Saw wood	Guide	
Put on glove	Tread on	
Open box	Drag	
Water plant	Help get up	
Lift table	Shout at	
Push shelf	Tie up	
Drag bag	Pinch ear	
Mix flour	Beckon	
Pick up basket	Brush	
Pour water	Scold	
Tear paper	Tickle	
Open umbrella	Bandage	
Hang up laundry	Scare	
Wipe bowl	Feed	
Saw wood	Guide	
Hammer nail	Fan	

## APPENDIX C: SENTENCE TYPES IN THE EVENT DESCRIPTION TASK

**Table C1.** Proportion of description types for transitive (two-participant) events in Experiment 1 and Experiment 2.

**Description type**	**Experiment 1**		**Experiment 2**	
	**Basque**	**Spanish**	**Basque**	**Spanish**
Canonical (Agent-Patient)	3679	3613	3040	3139
“Lisa has kicked Tim”	(97,2%)	(94,9%)	(95,8%)	(96,1%)
Deviating (total)	105	191	130	126
	(2,8%)	(5,1 %)	(4,2%)	(3,9%)
Reciprocal	37	90	89	76
“Lisa and Tim have danced”				
Other expressions	65	95	36	47
“Lisa appeared in front of Tim”				
Patient-initial	3	6	5	3
“Tim has been dragged by Lisa”				

## References

[bib1] Altmann, G. T. M. (2004). Language-mediated eye movements in the absence of a visual world: The ‘blank screen paradigm’. Cognition, 93(2), B79–B87. 10.1016/j.cognition.2004.02.005, 15147941

[bib2] Altmann, G. T. M., & Ekves, Z. (2019). Events as intersecting object histories: A new theory of event representation. Psychological Review, 126(6), 817–840. 10.1037/rev0000154, 31144837

[bib3] Anwyl-Irvine, A., Dalmaijer, E. S., Hodges, N., & Evershed, J. K. (2021). Realistic precision and accuracy of online experiment platforms, web browsers, and devices. Behavior Research Methods, 53(4), 1407–1425. 10.3758/s13428-020-01501-5, 33140376 PMC8367876

[bib4] Athanasopoulos, P., & Bylund, E. (2013). Does grammatical aspect affect motion event cognition? A cross-linguistic comparison of English and Swedish speakers. Cognitive Science, 37(2), 286–309. 10.1111/cogs.12006, 23094696

[bib5] Baddeley, A. (2007). Working memory, thought, and action. Oxford University Press. 10.1093/acprof:oso/9780198528012.001.0001

[bib6] Baddeley, A., Allen, R. J., & Hitch, G. J. (2011). Binding in visual working memory: The role of the episodic buffer. Neuropsychologia, 49(6), 1393–1400. 10.1016/j.neuropsychologia.2010.12.042, 21256143

[bib7] Baddeley, A., Hitch, G., & Allen, R. (2009). Working memory and binding in sentence recall. Journal of Memory and Language, 61(3), 438–456. 10.1016/j.jml.2009.05.004

[bib8] Baldassano, C., Hasson, U., & Norman, K. A. (2018). Representation of real-world event schemas during narrative perception. The Journal of Neuroscience, 38(45), 9689–9699. 10.1523/JNEUROSCI.0251-18.2018, 30249790 PMC6222059

[bib9] Barr, D. J. (2008). Analyzing ‘visual world’ eyetracking data using multilevel logistic regression. Journal of Memory and Language, 59(4), 457–474. 10.1016/j.jml.2007.09.002

[bib10] Bickel, B. (2010). Grammatical relations typology. In J. J. Song (Ed.), The Oxford handbook of linguistic typology (pp. 399–444). Oxford University Press. 10.1093/oxfordhb/9780199281251.013.0020

[bib11] Bickel, B., & Nichols, J. (2009). Case marking and alignment. In A. L. Malchukov & A. Spencer (Eds.), The Oxford handbook of case (pp. 304–321). Oxford University Press. 10.1093/oxfordhb/9780199206476.013.0021

[bib12] Bickel, B., Witzlack-Makarevich, A., Choudhary, K. K., Schlesewsky, M., & Bornkessel-Schlesewsky, I. (2015). The neurophysiology of language processing shapes the evolution of grammar: Evidence from case marking. PLOS ONE, 10(8), Article e0132819. 10.1371/journal.pone.0132819, 26267884 PMC4534460

[bib13] Blasi, D. E., Henrich, J., Adamou, E., Kemmerer, D., & Majid, A. (2022). Over-reliance on English hinders cognitive science. Trends in Cognitive Sciences, 26(12), 1153–1170. 10.1016/j.tics.2022.09.015, 36253221

[bib14] Bock, K., & Ferreira, V. S. (2014). Syntactically speaking. In M. Goldrick, V. S. Ferreira, & M. Miozzo (Eds.), The Oxford handbook of language production (pp. 21–46). Oxford University Press. 10.1093/oxfordhb/9780199735471.013.008

[bib15] Bresnan, J. (1982). The mental representation of grammatical relations. MIT Press.

[bib16] Bridges, D., Pitiot, A., MacAskill, M. R., & Peirce, J. W. (2020). The timing mega-study: Comparing a range of experiment generators, both lab-based and online. PeerJ, 8, Article e9414. 10.7717/peerj.9414, 33005482 PMC7512138

[bib17] Bürkner, P.-C. (2017). brms: An R package for Bayesian multilevel models using Stan. Journal of Statistical Software, 80(1), 1–28. 10.18637/jss.v080.i01

[bib18] Bürkner, P.-C. (2018). Advanced Bayesian multilevel modeling with the R package brms. The R Journal, 10(1), 395–411. 10.32614/RJ-2018-017

[bib19] Carpenter, B., Gelman, A., Hoffman, M. D., Lee, D., Goodrich, B., Betancourt, M., Brubaker, M., Guo, J., Li, P., & Riddell, A. (2017). Stan: A probabilistic programming language. Journal of Statistical Software, 76(1), 1–32. 10.18637/jss.v076.i01, 36568334 PMC9788645

[bib20] Carpenter, R. H. S., & Williams, M. L. L. (1995). Neural computation of log likelihood in control of saccadic eye movements. Nature, 377(6544), 59–62. 10.1038/377059a0, 7659161

[bib21] Cohn, N., & Paczynski, M. (2013). Prediction, events, and the advantage of Agents: The processing of semantic roles in visual narrative. Cognitive Psychology, 67(3), 73–97. 10.1016/j.cogpsych.2013.07.002, 23959023 PMC3895484

[bib22] de Bruin, A., Carreiras, M., & Duñabeitia, J. A. (2017). The BEST dataset of language proficiency. Frontiers in Psychology, 8, Article 522. 10.3389/fpsyg.2017.00522, 28428768 PMC5382213

[bib23] Demiral, Ş. B., Schlesewsky, M., & Bornkessel-Schlesewsky, I. (2008). On the universality of language comprehension strategies: Evidence from Turkish. Cognition, 106(1), 484–500. 10.1016/j.cognition.2007.01.008, 17336956

[bib24] Dobel, C., Glanemann, R., Kreysa, H., Zwitserlood, P., & Eisenbeiß, S. (2011). Visual encoding of coherent and non-coherent scenes. In J. Bohnemeyer & E. Pederson (Eds.), Event representation in language and cognition (Vol. 11, pp. 189–215). Cambridge University Press. 10.1017/CBO9780511782039.009

[bib25] Dobel, C., Gumnior, H., Bölte, J., & Zwitserlood, P. (2007). Describing scenes hardly seen. Acta Psychologica, 125(2), 129–143. 10.1016/j.actpsy.2006.07.004, 16934737

[bib26] Dowty, D. (1991). Thematic proto-roles and argument selection. Language, 67, 547–619. 10.1353/lan.1991.0021

[bib27] Dryer, M. S. (2013). Order of subject, object and verb. In M. S. Dryer & M. Haspelmath (Eds.), The world atlas of language structures online. Max Planck Institute for Evolutionary Anthropology.

[bib28] Duchowski, A. T. (2007). Eye tracking methodology: Theory and practice (2nd ed.). Springer. 10.1007/978-1-84628-609-4

[bib29] Egurtzegi, A., Blasi, D. E., Bornkessel-Schlesewsky, I., Laka, I., Meyer, M., Bickel, B., & Sauppe, S. (2022). Cross-linguistic differences in case marking shape neural power dynamics and gaze behavior during sentence planning. Brain and Language, 230, Article 105127. 10.1016/j.bandl.2022.105127, 35605312

[bib30] Ferreira, F., Apel, J., & Henderson, J. M. (2008). Taking a new look at looking at nothing. Trends in Cognitive Sciences, 12(11), 405–410. 10.1016/j.tics.2008.07.007, 18805041

[bib31] Ferreira, F., & Swets, B. (2002). How incremental is language production? Evidence from the production of utterances requiring the computation of arithmetic sums. Journal of Memory and Language, 46(1), 57–84. 10.1006/jmla.2001.2797

[bib32] Ferreira, V. S. (1996). Is it better to give than to donate? Syntactic flexibility in language production. Journal of Memory and Language, 35(5), 724–755. 10.1006/jmla.1996.0038

[bib33] Fillmore, C. (1968). The case for the case. In E. Bach & R. Harms (Eds.), Universals in linguistic theory. Rinehart and Winston.

[bib34] Firestone, C., & Scholl, B. J. (2016). Cognition does not affect perception: Evaluating the evidence for “top-down” effects. Behavioral and Brain Sciences, 39, Article e229. 10.1017/S0140525X15000965, 26189677

[bib35] Flecken, M., Gerwien, J., Carroll, M., & von Stutterheim, C. (2015). Analyzing gaze allocation during language planning: A cross-linguistic study on dynamic events. Language and Cognition, 7(1), 138–166. 10.1017/langcog.2014.20

[bib36] Frank, M. C., Vul, E., & Johnson, S. P. (2009). Development of infants’ attention to faces during the first year. Cognition, 110(2), 160–170. 10.1016/j.cognition.2008.11.010, 19114280 PMC2663531

[bib37] Galazka, M., & Nyström, P. (2016). Infants’ preference for individual agents within chasing interactions. Journal of Experimental Child Psychology, 147, 53–70. 10.1016/j.jecp.2016.02.010, 27017143

[bib38] Gervais, W. M., Reed, C. L., Beall, P. M., & Roberts, R. J. (2010). Implied body action directs spatial attention. Attention, Perception, & Psychophysics, 72(6), 1437–1443. 10.3758/APP.72.6.1437, 20675790

[bib39] Gerwien, J., & Flecken, M. (2016). First things first? Top-down influences on event apprehension. In A. Papafragou, D. Grodner, D. Mirman, & J. C. Trueswell (Eds.), Proceedings of the 38th Annual Meeting of the Cognitive Science Society (pp. 2633–2638). Cognitive Science Society.

[bib40] Gibson, E., Piantadosi, S. T., Brink, K., Bergen, L., Lim, E., & Saxe, R. (2013). A noisy-channel account of crosslinguistic word-order variation. Psychological Science, 24(7), 1079–1088. 10.1177/0956797612463705, 23649563

[bib41] Gilbert, C. D., & Li, W. (2013). Top-down influences on visual processing. Nature Reviews Neuroscience, 14(5), 350–363. 10.1038/nrn3476, 23595013 PMC3864796

[bib42] Gold, J. M., Murray, R. F., Sekuler, A. B., Bennett, P. J., & Sekuler, R. (2005). Visual memory decay is deterministic. Psychological Science, 16(10), 769–774. 10.1111/j.1467-9280.2005.01612.x, 16181438

[bib43] Goldin-Meadow, S., Chee So, W., Özyürek, A., & Mylander, C. (2008). The natural order of events: How speakers of different languages represent events nonverbally. Proceedings of the National Academy of Sciences of the United States of America, 105(27), 9163–9168. 10.1073/pnas.0710060105, 18599445 PMC2453738

[bib44] Greene, M. R., & Oliva, A. (2009). The briefest of glances: The time course of natural scene understanding. Psychological Science, 20(4), 464–472. 10.1111/j.1467-9280.2009.02316.x, 19399976 PMC2742770

[bib45] Griffin, Z. M., & Bock, K. (2000). What the eyes say about speaking. Psychological Science, 11(4), 274–279. 10.1111/1467-9280.00255, 11273384 PMC5536117

[bib46] Gruber, J. S. (1965). Studies in lexical relations [Unpublished doctoral dissertation]. Massachusetts Institute of Technology. (Reprinted as part of *Lexical Structures in Syntax and Semantics*, North-Holland, Amsterdam, 1976)

[bib47] Guterstam, A., & Graziano, M. S. A. (2020). Implied motion as a possible mechanism for encoding other people’s attention. Progress in Neurobiology, 190, Article 101797. 10.1016/j.pneurobio.2020.101797, 32217129

[bib48] Hafri, A., Papafragou, A., & Trueswell, J. C. (2013). Getting the gist of events: Recognition of two-participant actions from brief displays. Journal of Experimental Psychology: General, 142(3), 880–905. 10.1037/a0030045, 22984951 PMC3657301

[bib49] Hafri, A., Trueswell, J. C., & Strickland, B. (2018). Encoding of event roles from visual scenes is rapid, spontaneous, and interacts with higher-level visual processing. Cognition, 175, 36–52. 10.1016/j.cognition.2018.02.011, 29459238 PMC5879027

[bib50] Hall, M. L., Mayberry, R. I., & Ferreira, V. S. (2013). Cognitive constraints on constituent order: Evidence from elicited pantomime. Cognition, 129(1), 1–17. 10.1016/j.cognition.2013.05.004, 23792806 PMC4224279

[bib51] Haupt, F. S., Schlesewsky, M., Roehm, D., Friederici, A. D., & Bornkessel-Schlesewsky, I. (2008). The status of subject–object reanalyses in the language comprehension architecture. Journal of Memory and Language, 59(1), 54–96. 10.1016/j.jml.2008.02.003

[bib52] Henderson, J. M., Brockmole, J. R., Castelhano, M. S., & Mack, M. (2007). Visual saliency does not account for eye movements during visual search in real-world scenes. In R. P. G. van Gompel, M. H. Fischer, W. S. Murray, & R. L. Hill (Eds.), Eye movements: A window on mind and brain (pp. 537–562). Elsevier. 10.1016/B978-008044980-7/50027-6

[bib53] Henderson, J. M., Hayes, T. R., Rehrig, G., & Ferreira, F. (2018). Meaning guides attention during real-world scene description. Scientific Reports, 8, Article 13504. 10.1038/s41598-018-31894-5, 30202075 PMC6131246

[bib54] Henderson, J. M., Malcolm, G. L., & Schandl, C. (2009). Searching in the dark: Cognitive relevance drives attention in real-world scenes. Psychonomic Bulletin & Review, 16(5), 850–856. 10.3758/PBR.16.5.850, 19815788

[bib55] Henrich, J., Heine, S. J., & Norenzayan, A. (2010). The weirdest people in the world? Behavioral and Brain Sciences, 33(2–3), 61–135. 10.1017/S0140525X0999152X, 20550733

[bib56] Hesse, C., & Franz, V. H. (2010). Grasping remembered objects: Exponential decay of the visual memory. Vision Research, 50(24), 2642–2650. 10.1016/j.visres.2010.07.026, 20692279

[bib57] Hoyer, W. J., Stawski, R. S., Wasylyshyn, C., & Verhaeghen, P. (2004). Adult age and digit symbol substitution performance: A meta-analysis. Psychology and Aging, 19(1), 211–214. 10.1037/0882-7974.19.1.211, 15065945

[bib58] Huettig, F., & Janse, E. (2016). Individual differences in working memory and processing speed predict anticipatory spoken language processing in the visual world. Language, Cognition and Neuroscience, 31(1), 80–93. 10.1080/23273798.2015.1047459

[bib59] Ivanova, A. A., Mineroff, Z., Zimmerer, V., Kanwisher, N., Varley, R., & Fedorenko, E. (2021). The language network is recruited but not required for nonverbal event semantics. Neurobiology of Language, 2(2), 176–201. 10.1162/nol_a_00030 37216147 PMC10158592

[bib60] Kamide, Y., Scheepers, C., & Altmann, G. T. M. (2003). Integration of syntactic and semantic information in predictive processing: Cross-linguistic evidence from German and English. Journal of Psycholinguistic Research, 32(1), 37–55. 10.1023/A:1021933015362, 12647562

[bib61] Kemmerer, D. (2012). The cross-linguistic prevalence of SOV and SVO word orders reflects the sequential and hierarchical representation of action in Broca’s area. Language and Linguistics Compass, 6(1), 50–66. 10.1002/lnc3.322

[bib62] Kourtzi, Z., & Kanwisher, N. (2000). Activation in human MT/MST by static images with implied motion. Journal of Cognitive Neuroscience, 12(1), 48–55. 10.1162/08989290051137594, 10769305

[bib63] Krekelberg, B., Vatakis, A., & Kourtzi, Z. (2005). Implied motion from form in the human visual cortex. Journal of Neurophysiology, 94(6), 4373–4386. 10.1152/jn.00690.2005, 16107528

[bib64] Kruschke, J. K. (2015). Doing Bayesian data analysis: A tutorial with R, JAGS, and Stan (2nd ed.). Academic Press.

[bib65] Laka, I. (2006). Deriving split ergativity in the progressive: The case of Basque. In A. Johns, D. Massam, & J. Ndayiragije (Eds.), Ergativity: Emerging issues (Vol. 65, pp. 173–195). Springer. 10.1007/1-4020-4188-8_7

[bib66] Langacker, R. W. (2008). Cognitive grammar: A basic introduction. Oxford University Press. 10.1093/acprof:oso/9780195331967.001.0001

[bib67] Lenth, R. V. (2020). emmeans: Estimated marginal means, aka least-squares means (R package version 1.5.2-1) [Computer software manual]. https://cran.r-project.org/web/packages/emmeans/index.html

[bib68] Majid, A., & Levinson, S. C. (2010). WEIRD languages have misled us, too. Behavioral and Brain Sciences, 33(2–3), 103. 10.1017/S0140525X1000018X, 20546660

[bib69] Matzke, M., Mai, H., Nager, W., Rüsseler, J., & Münte, T. (2002). The costs of freedom: An ERP – study of non-canonical sentences. Clinical Neurophysiology, 113(6), 844–852. 10.1016/S1388-2457(02)00059-7, 12048043

[bib70] Meir, I., Aronoff, M., Börstell, C., Hwang, S.-O., Ilkbasaran, D., Kastner, I., Lepic, R., Lifshitz Ben-Basat, A., Padden, C., & Sandler, W. (2017). The effect of being human and the basis of grammatical word order: Insights from novel communication systems and young sign languages. Cognition, 158, 189–207. 10.1016/j.cognition.2016.10.011, 27837693

[bib71] Meyer, A. S., Sleiderink, A. M., & Levelt, W. J. M (1998). Viewing and naming objects: Eye movements during noun phrase production. Cognition, 66(2), B25–B33. 10.1016/S0010-0277(98)00009-2, 9677766

[bib72] Morales, J., Yudes, C., Gómez-Ariza, C. J., & Bajo, M. T. (2015). Bilingualism modulates dual mechanisms of cognitive control: Evidence from ERPs. Neuropsychologia, 66, 157–169. 10.1016/j.neuropsychologia.2014.11.014, 25448864

[bib73] Napoli, D. J., & Sutton-Spence, R. (2014). Order of the major constituents in sign languages: Implications for all language. Frontiers in Psychology, 5, Article 376. 10.3389/fpsyg.2014.00376, 24860523 PMC4026690

[bib74] New, J., Cosmides, L., & Tooby, J. (2007). Category-specific attention for animals reflects ancestral priorities, not expertise. Proceedings of the National Academy of Sciences of the United States of America, 104(42), 16598–16603. 10.1073/pnas.0703913104, 17909181 PMC2034212

[bib75] Norcliffe, E., & Konopka, A. E. (2015). Vision and language in cross-linguistic research on sentence production. In R. K. Mishra, N. Srinivasan, & F. Huettig (Eds.), Attention and vision in language processing (pp. 77–96). Springer. 10.1007/978-81-322-2443-3_5

[bib76] Norcliffe, E., Konopka, A. E., Brown, P., & Levinson, S. C. (2015). Word order affects the time course of sentence formulation in Tzeltal. Language, Cognition and Neuroscience, 30(9), 1187–1208. 10.1080/23273798.2015.1006238

[bib77] Nordlinger, R., Garrido Rodriguez, G., & Kidd, E. (2022). Sentence planning and production in Murrinhpatha, an Australian ‘free word order’ language. Language, 98(2), 187–220. 10.1353/lan.2022.0008

[bib78] Olguin, A., Cekic, M., Bekinschtein, T. A., Katsos, N., & Bozic, M. (2019). Bilingualism and language similarity modify the neural mechanisms of selective attention. Scientific Reports, 9(1), Article 8204. 10.1038/s41598-019-44782-3, 31160645 PMC6547874

[bib79] Peirce, J., Gray, J. R., Simpson, S., MacAskill, M., Höchenberger, R., Sogo, H., Kastman, E., & Lindeløv, J. K. (2019). PsychoPy2: Experiments in behavior made easy. Behavior Research Methods, 51(1), 195–203. 10.3758/s13428-018-01193-y, 30734206 PMC6420413

[bib80] Pierce, J. E., Clementz, B. A., & McDowell, J. E. (2019). Saccades: Fundamentals and neural mechanisms. In C. Klein & U. Ettinger (Eds.), Eye movement research: An introduction to its scientific foundations and applications (pp. 11–71). Springer. 10.1007/978-3-030-20085-5_2

[bib81] Pratt, J., Radulescu, P. V., Guo, R. M., & Abrams, R. A. (2010). It’s alive!: Animate motion captures visual attention. Psychological Science, 21(11), 1724–1730. 10.1177/0956797610387440, 20974713

[bib82] Rad, M. S., Martingano, A. J., & Ginges, J. (2018). Toward a psychology of *Homo sapiens*: Making psychological science more representative of the human population. Proceedings of the National Academy of Sciences of the United States of America, 115(45), 11401–11405. 10.1073/pnas.1721165115, 30397114 PMC6233089

[bib83] Radvansky, G. A., & Zacks, J. M. (2011). Event perception: Event perception. Wiley Interdisciplinary Reviews: Cognitive Science, 2(6), 608–620. 10.1002/wcs.133, 23082236 PMC3472805

[bib84] R Core Team. (2022). R: A language and environment for statistical computing [Computer software manual]. R Foundation for Statistical Computing. Retrieved from https://www.R-project.org.

[bib85] Rehrig, G., Hayes, T. R., Henderson, J. M., & Ferreira, F. (2020). When scenes speak louder than words: Verbal encoding does not mediate the relationship between scene meaning and visual attention. Memory & Cognition, 48(7), 1181–1195. 10.3758/s13421-020-01050-4, 32430889 PMC8843103

[bib86] Richmond, L. L., & Zacks, J. M. (2017). Constructing experience: Event models from perception to action. Trends in Cognitive Sciences, 21(12), 962–980. 10.1016/j.tics.2017.08.005, 28899609 PMC5694361

[bib87] Rissman, L., & Majid, A. (2019). Thematic roles: Core knowledge or linguistic construct? Psychonomic Bulletin & Review, 26, 1850–1869. 10.3758/s13423-019-01634-5, 31290008 PMC6863944

[bib88] Roeser, J., Torrance, M., & Baguley, T. (2019). Advance planning in written and spoken sentence production. Journal of Experimental Psychology: Learning, Memory, and Cognition, 45(11), 1983–2009. 10.1037/xlm0000685, 30702314

[bib89] Salthouse, T. A. (2000). Aging and measures of processing speed. Biological Psychology, 54(1–3), 35–54. 10.1016/S0301-0511(00)00052-1, 11035219

[bib90] Santesteban, M., Pickering, M. J., Laka, I., & Branigan, H. P. (2015). Effects of case-marking and head position on language production? Evidence from an ergative OV language. Language, Cognition and Neuroscience, 30(9), 1175–1186. 10.1080/23273798.2015.1065335

[bib91] Sassenhagen, J., & Alday, P. M. (2016). A common misapplication of statistical inference: Nuisance control with null-hypothesis significance tests. Brain and Language, 162, 42–45. 10.1016/j.bandl.2016.08.001, 27543688

[bib92] Sauppe, S. (2016). Verbal semantics drives early anticipatory eye movements during the comprehension of verb-initial sentences. Frontiers in Psychology, 7, Article 95. 10.3389/fpsyg.2016.00095, 26903903 PMC4746280

[bib93] Sauppe, S. (2017). Word order and voice influence the timing of verb planning in German sentence production. Frontiers in Psychology, 8, Article 1648. 10.3389/fpsyg.2017.01648, 29018379 PMC5623055

[bib94] Sauppe, S., Choudhary, K. K., Giroud, N., Blasi, D. E., Norcliffe, E., Bhattamishra, S., Gulati, M., Egurtzegi, A., Bornkessel-Schlesewsky, I., Meyer, M., & Bickel, B. (2021). Neural signatures of syntactic variation in speech planning. PLOS Biology, 19(1), Article e3001038. 10.1371/journal.pbio.3001038, 33497384 PMC7837500

[bib95] Sauppe, S., & Flecken, M. (2021). Speaking for seeing: Sentence structure guides visual event apprehension. Cognition, 206, Article 104516. 10.1016/j.cognition.2020.104516, 33228969

[bib96] Sauppe, S., Norcliffe, E. J., Konopka, A. E., Van Valin, R. D., & Levinson, S. C. (2013). Dependencies first: Eye tracking evidence from sentence production in Tagalog. In M. Knauff, M. Pauen, N. Sebanz, & I. Wachsmuth (Eds.), Proceedings of the 35th Annual Conference of the Cognitive Science Society (pp. 1265–1270). Cognitive Science Society.

[bib97] Schneider, W., Eschman, A., & Zuccolotto, A. (2002). E-Prime reference guide. Psychology Software Tools Inc.

[bib98] Schouwstra, M., & de Swart, H. (2014). The semantic origins of word order. Cognition, 131(3), 431–436. 10.1016/j.cognition.2014.03.004, 24704967

[bib99] Slobin, D. I. (1987). Thinking for speaking. Proceedings of the 13th Annual Meeting of the Berkeley Linguistics Society, 13, 435–445. 10.3765/bls.v13i0.1826

[bib100] Spelke, E. S., & Kinzler, K. D. (2007). Core knowledge. Developmental Science, 10(1), 89–96. 10.1111/j.1467-7687.2007.00569.x, 17181705

[bib101] Stallings, L. M., MacDonald, M. C., & O’Seaghdha, P. G. (1998). Phrasal ordering constraints in sentence production: Phrase length and verb disposition in heavy-NP shift. Journal of Memory and Language, 39(3), 392–417. 10.1006/jmla.1998.2586

[bib102] Staudte, M., & Altmann, G. T. M. (2017). Recalling what was where when seeing nothing there. Psychonomic Bulletin & Review, 24(2), 400–407. 10.3758/s13423-016-1104-8, 27432003 PMC5390004

[bib103] Stawarczyk, D., Bezdek, M. A., & Zacks, J. M. (2021). Event representations and predictive processing: The role of the midline default network core. Topics in Cognitive Science, 13(1), 164–186. 10.1111/tops.12450, 31486286 PMC7984453

[bib104] Stoet, G. (2010). PsyToolkit: A software package for programming psychological experiments using Linux. Behavior Research Methods, 42(4), 1096–1104. 10.3758/BRM.42.4.1096, 21139177

[bib105] Stoet, G. (2017). PsyToolkit: A novel web-based method for running online questionnaires and reaction-time experiments. Teaching of Psychology, 44(1), 24–31. 10.1177/0098628316677643

[bib106] Summerfield, C., & de Lange, F. P. (2014). Expectation in perceptual decision making: Neural and computational mechanisms. Nature Reviews Neuroscience, 15(11), 745–756. 10.1038/nrn3838, 25315388

[bib107] Vandenbroucke, A. R., Sligte, I. G., & Lamme, V. A. (2011). Manipulations of attention dissociate fragile visual short-term memory from visual working memory. Neuropsychologia, 49(6), 1559–1568. 10.1016/j.neuropsychologia.2010.12.044, 21236273

[bib108] van de Velde, M., Meyer, A. S., & Konopka, A. E. (2014). Message formulation and structural assembly: Describing “easy” and “hard” events with preferred and dispreferred syntactic structures. Journal of Memory and Language, 71(1), 124–144. 10.1016/j.jml.2013.11.001

[bib109] Van Valin, R. D. (2006). Semantic macroroles and language processing. In I. Bornkessel, Schlesewsky M., B. Comrie, & A. D. Friederici (Eds.), Semantic role universals and argument linking: Theoretical, typological, and psycholinguistic perspectives (pp. 263–301). Mouton de Gruyter. 10.1515/9783110219272.263

[bib110] Verfaillie, K., & Daems, A. (1996). The priority of the agent in visual event perception: On the cognitive basis of grammatical agent-patient asymmetries. Cognitive Linguistics, 7(2), 131–148. 10.1515/cogl.1996.7.2.131

[bib111] Wagner, V., Jescheniak, J. D., & Schriefers, H. (2010). On the flexibility of grammatical advance planning during sentence production: Effects of cognitive load on multiple lexical access. Journal of Experimental Psychology: Learning, Memory, and Cognition, 36(2), 423–440. 10.1037/a0018619, 20192540

[bib112] Wang, L., Schlesewsky, M., Bickel, B., & Bornkessel-Schlesewsky, I. (2009). Exploring the nature of the ‘subject’-preference: Evidence from the online comprehension of simple sentences in Mandarin Chinese. Language and Cognitive Processes, 24, 1180–1226. 10.1080/01690960802159937

[bib113] Wechsler, D. A. (1997). Wechsler adult intelligence scale—3rd edition (No. 9). Psychological Corporation. 10.1037/t49755-000

[bib114] Wilson, F., Papafragou, A., Bunger, A., & Trueswell, J. (2011). Rapid extraction of event participants in caused motion events. In Proceedings of the 33rd Annual Conference of the Cognitive Science Society (pp. 1206–1211). Cognitive Science Society.

[bib115] Wilson, V. A. D., Zuberbühler, K., & Bickel, B. (2022). The evolutionary origins of syntax: Event cognition in nonhuman primates. Science Advances, 8(25), Article eabn8464. 10.1126/sciadv.abn8464, 35731868 PMC9216513

[bib116] Wolfe, J. M., & Horowitz, T. S. (2017). Five factors that guide attention in visual search. Nature Human Behaviour, 1(3), Article 0058. 10.1038/s41562-017-0058, 36711068 PMC9879335

[bib117] Yow, W. Q., & Li, X. (2015). Balanced bilingualism and early age of second language acquisition as the underlying mechanisms of a bilingual executive control advantage: Why variations in bilingual experiences matter. Frontiers in Psychology, 6, Article 164. 10.3389/fpsyg.2015.00164, 25767451 PMC4341428

[bib118] Zacks, J. M. (2020). Event perception and memory. Annual Review of Psychology, 71(1), 165–191. 10.1146/annurev-psych-010419-051101, 31905113 PMC8679009

[bib119] Zacks, J. M., Speer, N. K., Swallow, K. M., Braver, T. S., & Reynolds, J. R. (2007). Event perception: A mind-brain perspective. Psychological Bulletin, 133(2), 273–293. 10.1037/0033-2909.133.2.273, 17338600 PMC2852534

[bib120] Ünal, E., Ji, Y., & Papafragou, A. (2021). From event representation to linguistic meaning. Topics in Cognitive Science, 13(1), 224–242. 10.1111/tops.12475, 31692213

[bib121] Ünal, E., Richards, C., Trueswell, J. C., & Papafragou, A. (2021). Representing agents, patients, goals and instruments in causative events: A cross-linguistic investigation of early language and cognition. Developmental Science, 24(6), Article e13116. 10.1111/desc.13116, 33955664

